# Adsorption and Corrosion Performance of New Cationic Gemini Surfactants Derivatives of Fatty Amido Ethyl Aminium Chloride with Ester Spacer for Mild Steel in Acidic Solutions

**DOI:** 10.3390/ma13122790

**Published:** 2020-06-20

**Authors:** Nashwa S. Bin-Hudayb, Entsar E. Badr, M.A. Hegazy

**Affiliations:** 1 Department of Chemistry, College of Science, Qassim University, 52318 Qassim, Saudi Arabia; 371217399@qu.edu.sa; 2 Department of Chemistry, Faculty of Science Girls Branch, Al-Azhar University, 11754 Cairo, Egypt; 3 Egyptian Petroleum Research Institute (EPRI), Nasr, 11727 Cairo, Egypt

**Keywords:** cationic gemini surfactants, mild steel, inhibition, EIS, Tafel, weight loss

## Abstract

Three new cationic gemini surfactants with ester spacer type 2-2′-(ethane-1,2-diyl bis(oxy)) bis(N-(2-alkanamidoethyl)-N,N-dimethyl-2-oxoethan-1-aminium)) dichloride) (CGSES12, CGSES14 and CGSES16), based on *N*,*N*-dimethyl fatty amido ethylamine, were produced. These gemini quaternary ammonium salts were synthesized using a three-step reaction method, starting from th/e condensation of the fatty acid chloride (RCOCl) of various hydrophobic chain lengths (R, C_11_H_23_, C_13_H_27_, C_15_H_31_) with *N*,*N*-dimethyl ethylene diamine, followed by the quaternization of the tertiary amino group formed with the spacer of the ester group formed in the second step. The chemical configuration of the surfactants was established by FT-IR, ^1^HNMR, ^13^CNMR and Mass spectroscopies. The inhibition performance of three surfactants was studied by weight loss and electrochemical measurements. The results show that CGSES12, CGSES14 and CGSES16 behave as effective inhibitors and surface agents. The maximum efficiency was higher than 94% at 2.5 mM, and the inhibition order was CGSES16 > CGSES14 > CGSES12. This was due to the increment in hydrophobicity of the gemini surfactants. Their adsorption on a mild steel surface followed the Langmuir isotherm. CGSES12, CGSES14 and CGSES16 can be considered mixed-type inhibitors. The presence of CGSES12, CGSES14 and CGSES16 increased charge transfer resistance and decreased the corrosion rate. The adsorption focused on heteroatoms and the surface properties of cationic gemini surfactants.

## 1. Introduction

In the petroleum industry, mild steel (MS) is used in production pipelines, flooding systems, storage tanks, production stations, heat exchangers, and so on as a construction material [1,2,3]. MS is chosen for these applications due to its mechanical properties and cheap price compared to other metals like copper, aluminum, etc. [4,5,6]. But the main problem in using MS in the industry is corrosion, especially in industrial processes that used acids for, e.g., industrial cleaning or the acidization of petroleum wells [7,8]. The cost of metallic loss treatment adds to production expenses. Theref7*ore, the protection of MS against metallic loss is important to reduce costs [9,10]. The application of cationic surfactants as corrosion inhibitors decreases the economic burden of mild steel corrosion [11,12,13,14,15]. A literature review shows that cationic surfactants are added in small quantities to acidic solutions to inhibit MS dissolution [16,17,18]. The cost of treatment with surfactants is much lower than that of ordinary organic compounds [19,20,21] because the surfactant molecules move completely from the bulk of the solution to interface between MS and the acidic media when their concentration is less than the critical micelle concentration [22,23,24]. 

The objective of this study is the synthesis of new cationic gemini surfactants derivatives of fatty amido ethyl aminium chloride with ester spacers, and their application as corrosion inhibitors for MS in 1 M HCl. The structures of the three synthesized cationic gemini surfactants, namely 2-2′-(ethane-1,2-diyl bis(oxy)) bis(N-(2-dodecanamidoethyl)-N,N-dimethyl-2-oxoethan-1-aminium)) dichloride (CGSES12), 2-2′-(ethane-1,2-diyl bis(oxy)) bis(N-(2-tetradecanamidoethyl)-N,N-dimethyl-2-oxoethan-1-aminium)) dichloride (CGSES14) and 2-2′-(ethane-1,2-diyl bis(oxy))bis(N-(2-hexadecanamidoethyl)-N,N-dimethyl-2-oxoethan-1-aminium)) dichloride (CGSES16), were elucidated by FT-IR, ^1^HNMR, ^13^CNMR and Mass spectroscopies. Their inhibition effect was examined by gravimetric (weight loss), electrochemical impedance spectroscopy (EIS) and potentiodynamic polarization (PP) analysis. The adsorption thermodynamics of the inhibitors and the activation energy of MS dissolution were calculated and discussed to get information regarding corrosion inhibition based on the chemical structure of the surfactants.

## 2. Materials and Methods

### 2.1. Synthesis 

**Scheme 1:**
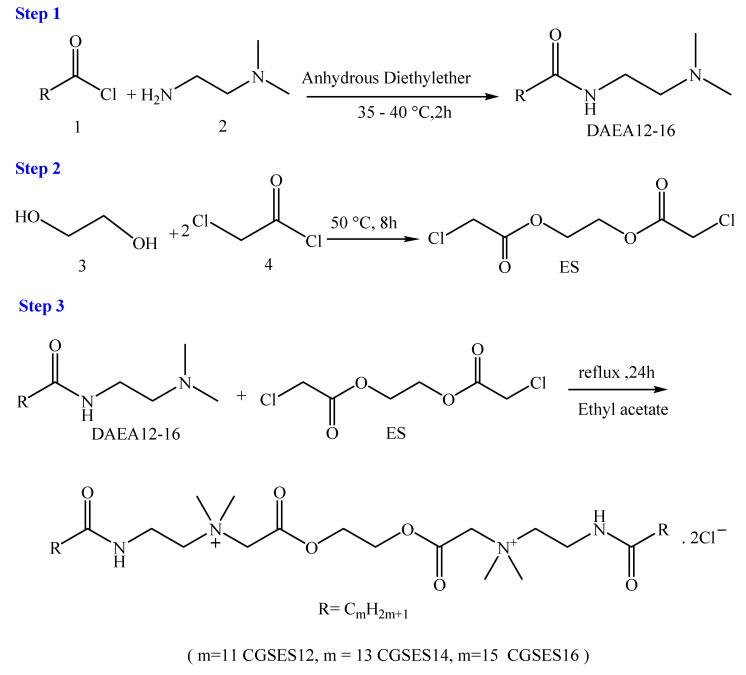
Synthesis of cationic gemini surfactants containing amide and ester group (CGSES12, CGSES14 and CGSES16).

The three-step method for the synthesis of the gemini surfactants with ester spacer (CGSES12, CGSES14, and CGSES16) is described in Scheme 1.

- Synthesis of N-(2-(dimethylamino)ethyl)dodecanamide (DAEA12)

Dodecanoyl chloride (18.17 g, 0.0831 mol) was added to a solution of *N*,*N*-dimethylethylendiamine **2** (24.24 g, 0.275 mol) in 150 mL anhydrous diethyl ether at 35–40 °C. The reaction was agitated for 2 h; then, the solvent was removed under reduced pressure. The subsequent product was washed with water, filtered off as a white solid and recrystallized using absolute ethanol. The final result was vacuum-dried [25].

Tetradecanoyl and Hexadecanoyl derivatives DAEA14; 16 were prepared similarly.

- Synthesis of the ester spacer (Ethane-1,2-diyl bis(2-chloroethanoate) (ES)
  The Gao method was used to synthesize ethane-1,2-diyl bis(2-chloroethanoate) [26].
- Synthesis of 2-2′-(Ethane-1,2-diylbis(oxy)) bis(N-(2-alkanamidoethyl)-N,N’-dimethyl-2-oxoethan-1-aminium)) dichloride (CGSES12-CGSES16)

To a round-bottomed flask equipped with magnetic stirring and a condenser was added (0.021 mol) *N*-(2-(dimethylamino) ethyl) dodecanamide; *N*-(2-(dimethylamino) ethyl) tetradecanamide or N-(2-(dimethylamino) ethyl) hexadecanamide and Ethane-1,2-diyl bis(2-chloroethanoate (2.14 g, 0.01 mol). Ethyl acetate was then added. The solution was heated to the reflux and the reaction was performed for 24 h. The obtained product was recrystallized from mixtures of ethyl acetate and ethanol (5:1 *v*/*v*). The finished product was vacuum-dried [26,27].

### 2.2. Specimens

The chemical compositions of the MS samples (wt%) are as follows: 0.19% C, 0.014% Ni, 0.003% Ti, 0.009% Cr, 0.022% Cu, 0.016% V, 0.05% Si, 0.009% P, 0.94% Mn, 0.004% S, 0.034% Al; the rest is Fe, as determined using an ARL™ 4460 Optical Emission 8 Spectrometer (Waltham, Middlesex,, Massachusetts USA). MS samples were abraded with emery papers with dissimilar grades (600–1200), and cleaned with acetone and distilled water.

### 2.3. Electrochemical Techniques

The electrochemical curves were determined using an OrigaLys device (OrigaLys ElectroChem SAS, 69140 Rillieux-la-Pape, Lyon, France), and analyzed in the OrigaMaster 5 software (OrigaLys ElectroChem SAS, 69140 Rillieux-la-Pape, Lyon France). An electrochemical cell contains a working electrode (WE), a saturated calomel electrode (SCE) as a reference electrode, and a platinum counter electrode (CE). The WE used in this study was MS fixed in a PVC vessel using epoxy resin; the uncovered area of the electrode in the solution was 0.34 cm^2^. First, the WE was submerged in a testing solution with an open circuit potential (OCP) for 30 min, until a stable state was attained. Potentiodynamic polarization (PP) measurements were implemented in a potential range from −800 to −300 mV vs. SCE at OCP with a scan rate of 0.2 mV s^−1^ at 20 °C. Electrochemical impedance spectroscopy (EIS) measurements were made in the frequency range of 100 kHz–30 mHz at a small alternating voltage perturbation (10 mV) at 20 °C.

### 2.4. Gravimetric Measurements

MS samples (2 cm × 6 cm × 0.6 cm) were soaked in 1 M HCl solution for one day in the absence and presence of numerous concentrations of CGSES12, CGSES14 and CGSES16. Then, the weight losses were measured and reported.

### 2.5. Surface Tension Measurements

A surface tension test was carried out as previously described [28,29,30]. The procedure is described in detail in the Supplementary Materials.

## 3. Results and Discussion

A series of three cationic gemini surfactants with ester spacer (CGSES12–16) was produced by acylation of the primary amino group *N*,*N*-dimethyl ethylenediamine with fatty acyl chloride using anhydrous diethyl ether as a solvent (Scheme 1). The corresponding N-(2 (dimethylamino)ethyl) alkanamide (DAEA12-16) intermediates were stirred independently with Ethane-1,2-diyl bis(2-chloroethanoate) (ES) to produce the desired surfactants after 24 h.

### 3.1. Construction Explanation

All synthesized cationic surfactants structures (CGSES12-16) were established using MS, NMR and FTIR, and the corresponding intermediates.

**Figure 1:**
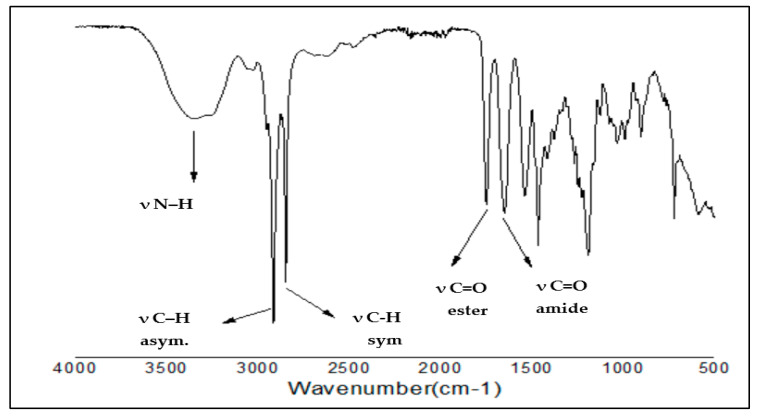
FT-IR spectrum for CGSES14.

The FTIR spectra of the intermediates (DAEA12-16) showed a strong absorption band at 1634–1643 cm^−1^, which is characteristic for C=O amide, and a sharp band in the range of 3291–3298 cm^−1^ for NH stretching. In addition to the distinct intermediate peaks (DAEA12-16), there was a strong peak at 1740–1755 cm^−1^ which corresponds to the absorption of C=O of the ester group for CGSES12-16 (Table 1, Figure 1). 

**Figure 2:**
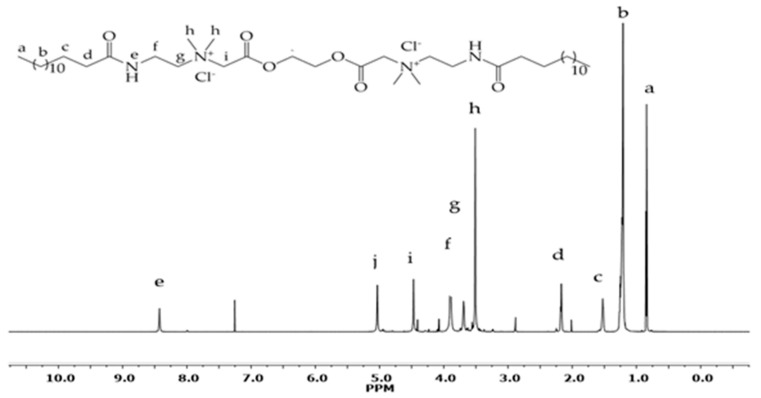
^1^H-NMR spectrum CGSES14.

The structure of CGSES14 is given as an example here. Providing the ^1^H NMR data for CGSES14 (Table 2, Figure 2), chemical shifts for methyl and methylene protons CH_2_ of the hydrophobic tail of the cationic gemini surfactants occurred at 0.86 and 1.20 ppm, respectively. A methylene proton signal before the carbonyl group, which is a part of the fatty chain, was seen at δ 2.19 ppm. The resonance of two methyl protons directly bound to the positive charge quaternary nitrogen [N^+^(CH_3_)_2_] was detected as a singlet at δ 3.57 ppm. The peaks at 3.92 and 3.72 ppm could be attributed to the methylene protons between the two nitrogen atoms, i.e., N- CH_2_ - CH_2_-N^+^. A typical resonance signal for the hydrogen protons of the methylene group directly bound to the positively charged quaternary nitrogen, which is a part of ester spacer, was identified from δ 4.49 ppm. The methylene proton signal next to the ester group, which is part of the spacer, was detected at δ 5.01 ppm. The resonance of one proton attached to nitrogen was shifted to the up field, and was affected by the ester group of the spacer, which was observed as a singlet at 8.45 ppm.

Also, the mass spectra data obtained for all of the studied current cationic gemini surfactants confirmed the chemical composition. The calculated molecular weight m/z values for cationic gemini surfactants (755.948, 812.057, 868.164,) perfectly matched the practical values (755, 812, 868) for CGSES12, CGSES14 and CGSES16 respectively (Table 3, Figure 3). The spectral data figures of the other synthesized cationic gemini surfactants with ester spacer are also provided in ESI (Figures S1–S14).

### 3.2. Potentiodynamic Polarization

**Figure 4:**
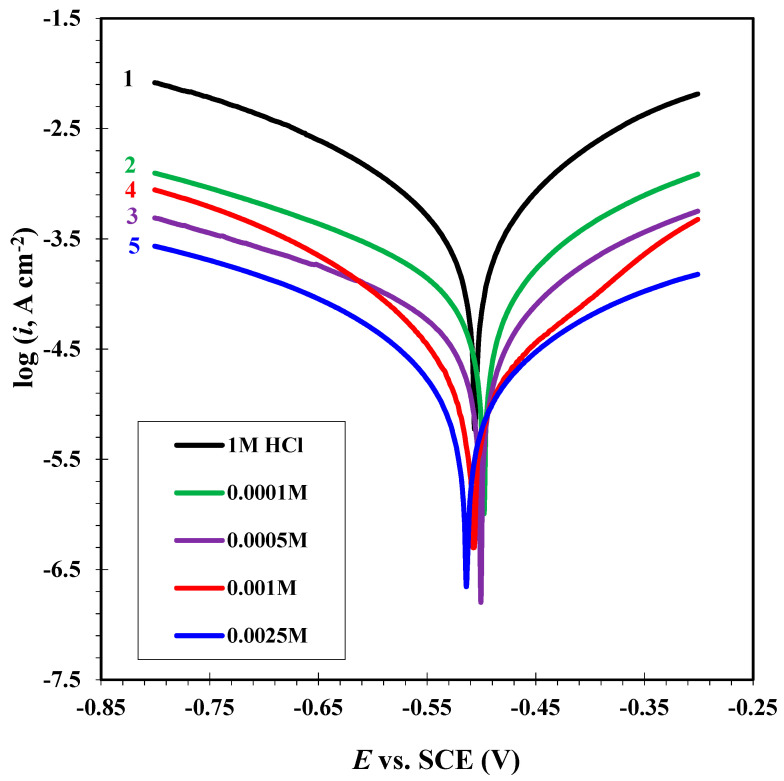
Anodic and cathodic polarization curves for MS obtained at 20 °C in 1 M HCl solution in the absence and presence of different concentrations of CGSES12.

**Figure 5:**
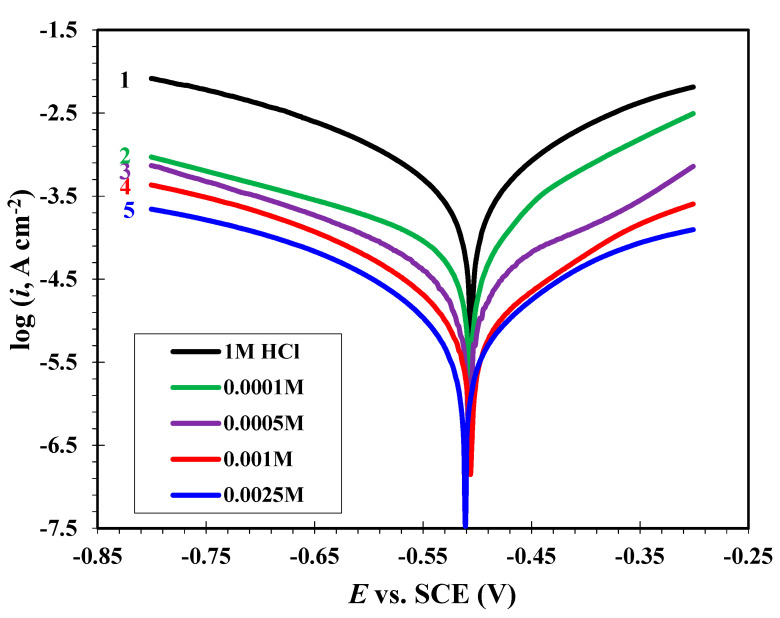
Anodic and cathodic polarization curves for MS obtained at 20 °C in 1 M HCl solution in the absence and presence of different concentrations of CGSES14.

**Figure 6:**
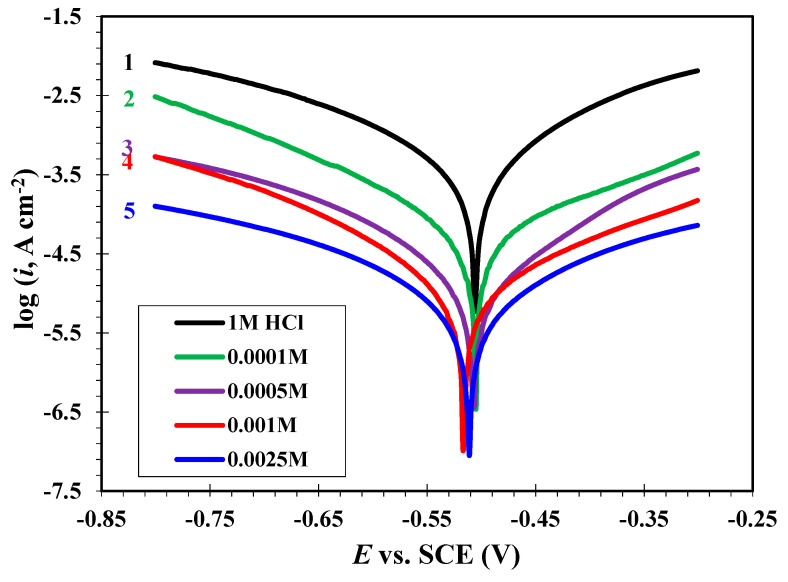
Anodic and cathodic polarization curves for MS obtained at 20 °C in 1 M HCl solution in the absence and presence of different concentrations of CGSES16.

Figure 4, Figure 5 and Figure 6 show the Tafel curves of MS in 1 M HCl in the absence and presence of dissimilar concentrations (0.0001, 0.005, 0.001 and 0.0025 M) of CGSES12, CGSES14 and CGSES16 at 20 °C. The addition of CGSES12, CGSES14, or CGSES16 moved the anodic and cathodic curves to a lower current density. This indicated that the corrosive anodic and cathodic reactions of MS were significantly inhibited by CGSES12, CGSES14 or CGSES16. Also, the Tafel curves match with each other. This indicates the activated control of both cathodic hydrogen permission and anodic ferrous dissolution. The corrosion parameters, like corrosion current density (*i*_corr_), corrosion potential (*E*_corr_), anodic and cathodic Tafel slopes (*β*_a_ and *β*_c_), were calculated using the OrigaMaster 5 software and are listed in Table 4. From the data in Table 4, it can be seen that *i*_corr_ decreased after adding CGSES12, CGSES14 or CGSES16 to 1 M HCl; it decreased gradually with greater CGSES12, CGSES14 or CGSES16 concentrations. This indicates that CGSES12, CGSES14 and CGSES16 are effective corrosion inhibitors. The corrosion inhibition efficiency (IE) was calculated as follows [31,32]:

(1) $$IE=\frac{i_{\mathrm{corr}}-{i_{\mathrm{corr}}}^{\left(\mathrm{inh}\right)}}{i_{\mathrm{corr}}}\times 100$$

where the corrosion current densities for MS in the uninhibited and inhibited solutions are represented by *i*_corr_ and *i*_corr_(inh), respectively.

The best inhibition efficiencies were 92.14, 95.77 and 96.40% for CGSES12, CGSES14 and CGSES16, respectively. This indicates that the inhibition efficiency increases with increasing the hydrophobic part (fatty alkyl chain) of the tested compounds.

*E*_corr_ is slightly changed in the presence of CGSES12, CGSES14 and CGSES16 for 1 M HCl, indicating that CGSES12, CGSES14 and CGSES16 are mixed-type inhibitors. Moreover, the electrochemical mechanisms of CGSES12, CGSES14 and CGSES16 may be attributed to the blocking effect [33]. The Tafel slopes (*β*_a_ and *β*_c_) changed slightly after the addition of CGSES12, CGSES14 and CGSES16 to a blank solution, indicating that CGSES12, CGSES14 and CGSES16 block the anodic and cathodic sites of the MS surface without affecting the dissolution reaction mechanism. 

### 3.3. EIS Results

**Figure 7:**
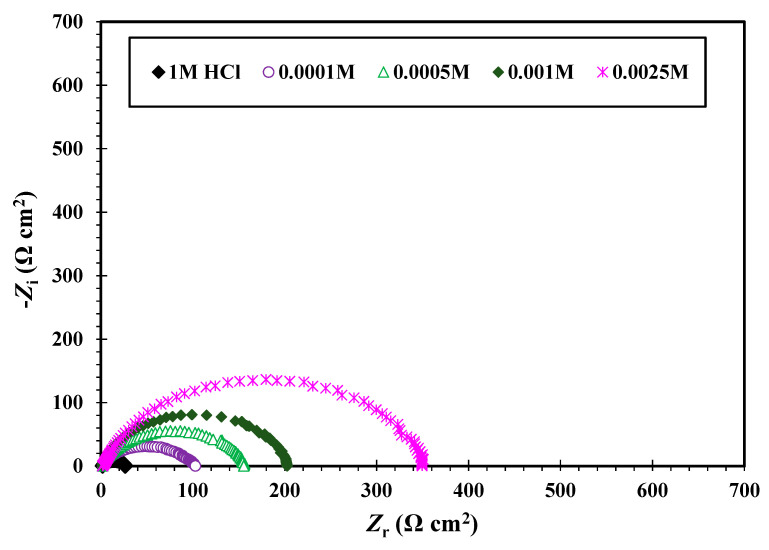
Nyquist plots for MS in 1 M HCl in the absence and presence of different concentrations of CGSES12 at 20 °C.

**Figure 8:**
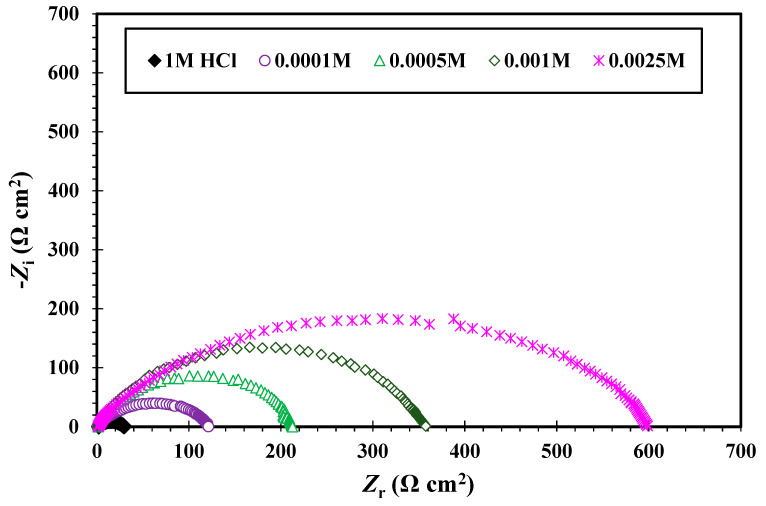
Nyquist plots for MS in 1 M HCl in the absence and presence of different concentrations of CGSES14 at 20 °C.

**Figure 9:**
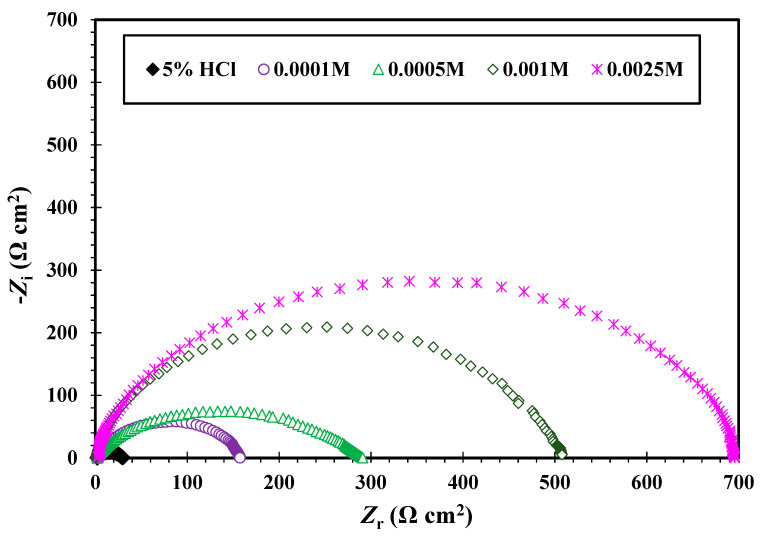
Nyquist plots for MS in 1 M HCl in the absence and presence of different concentrations of CGSES16 at 20 °C.

The Nyquist spectra of MS in 1 M HCl without and with of different concentrations (0.0001, 0.0005, 0.001 and 0.0025 M) of CGSES12, CGSES14 and CGSES16 are shown in Figure 7, Figure 8 and Figure 9. The diameter of the capacitive loop was higher in the presence CGSES12, CGSES14 and CGSES16. In addition, the diameter of the semicircle for the charge transfer resistance (*R*_ct_) increased with an increase in the concentrations of CGSES12, CGSES14 and CGSES16 from 0.0001 M to 0.0025 M. This indicates that these compounds adsorbed on the MS surface by blocking the active sites [34]. These spectra exhibited one single loop, indicating that the MS corrosion in 1 M HCl was controlled by the charge transfer process [35].

**Figure 10:**
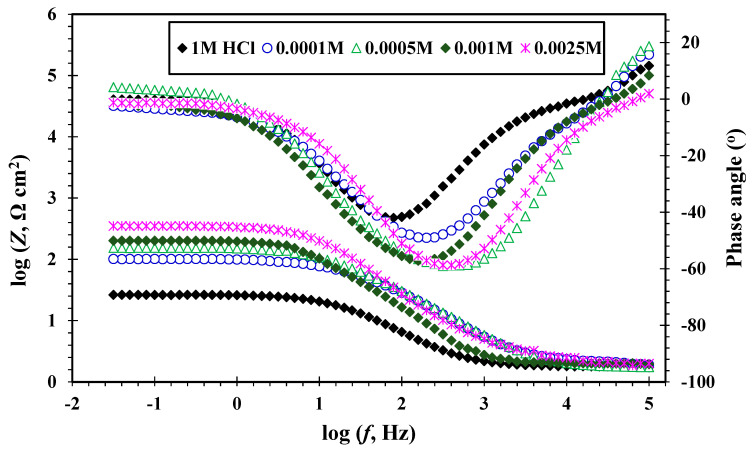
Bode plots for MS in 1 M HCl in the absence and presence of different concentrations of CGSES12 at 20.

**Figure 11:**
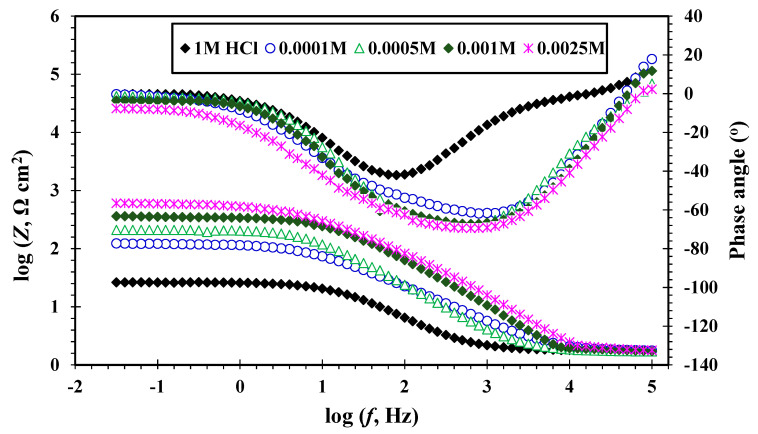
Bode plots for MS in 1 M HCl in the absence and presence of different concentrations of CGSES14 at 20 °C.

**Figure 12:**
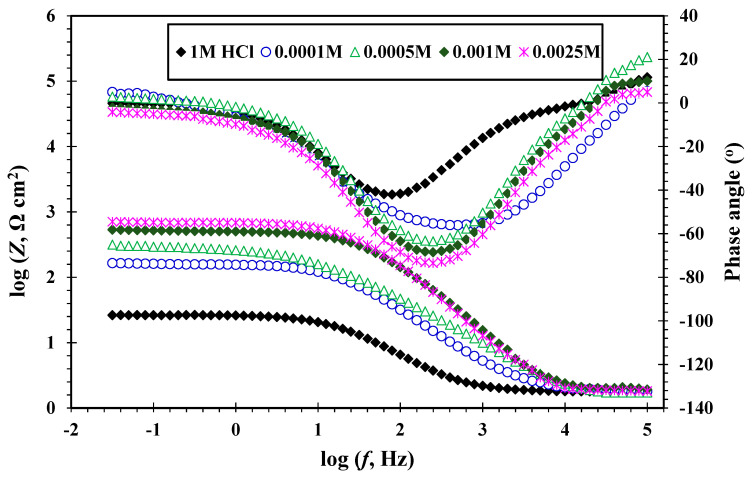
Bode plots for MS in 1 M HCl in the absence and presence of different concentrations of CGSES16 at 20 °C.

Figure 10, Figure 11 and Figure 12 show the Bode and Phase angle plots for MS without and with CGSES12, CGSES14 and CGSES16 in 1 M HCl. From these figures, we can see that the impedance value of the MS electrode in the bank solution was reduced in the presence of CGSES12, CGSES14 and CGSES16. The absolute impedance increased with increasing the concentration of CGSES12, CGSES14 and CGSES16. This means that CGSES12, CGSES14 and CGSES16 slow the ferrous dissolution and H_2_ evolution processes at the MS surface. Figure 10, Figure 11 and Figure 12 show a one phase peak which indicates that an one time constant related to the capacitive loop was present. We noted that the Nyquist and Bode shapes were the same after adding various concentrations of CGSES12, CGSES14 and CGSES16. This means that the mechanism of MS dissolution and hydrogen gas evolution in 1 M HCl was unmodified after adding CGSES12, CGSES14 and CGSES16. Also, the Nyquist plots were not perfect semicircles, and the phase angles were below 90°. This was the result of the roughness and inhomogeneity of the MS surface. This behavior was due to the frequency dispersion at a low frequency, leading to the deviation from an ideal electrical capacitance of the double layer ($C_{\mathrm{dl}}$) [36,37,38]. In this case, the constant phase element (CPE) is used instead of $C_{\mathrm{dl}}$, because CPE is more accurate [39,40].

**Figure 13:**
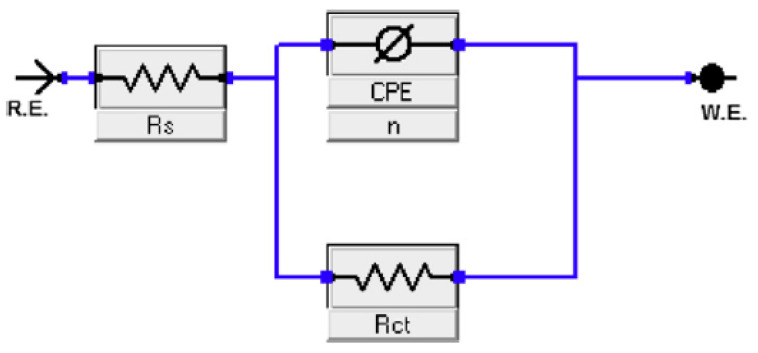
The suggested equivalent circuit model for the studied system.

**Figure 14:**
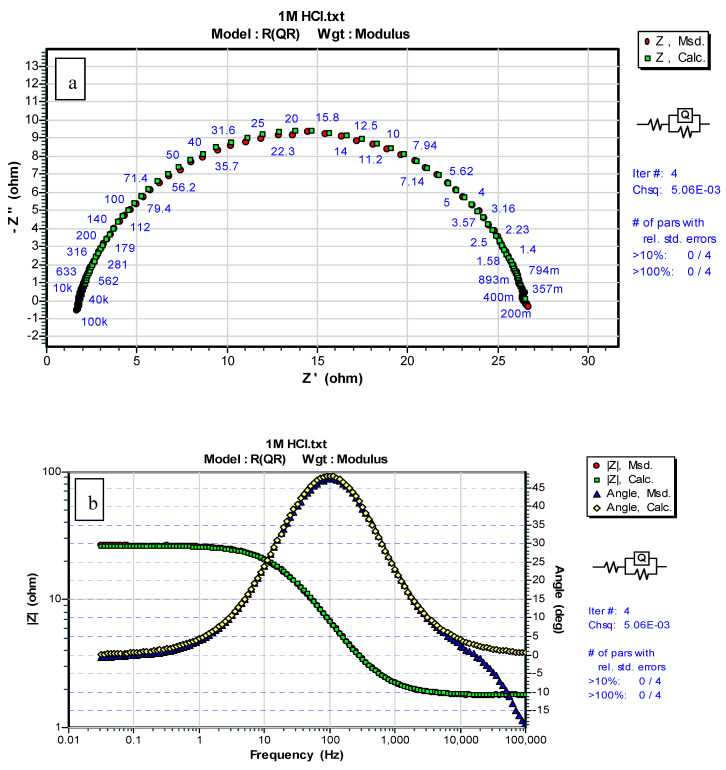
Simulation of EIS (**a**) and Bode (**b**) plots for MS in 1 M HCl at 20 °C.

**Figure 15:**
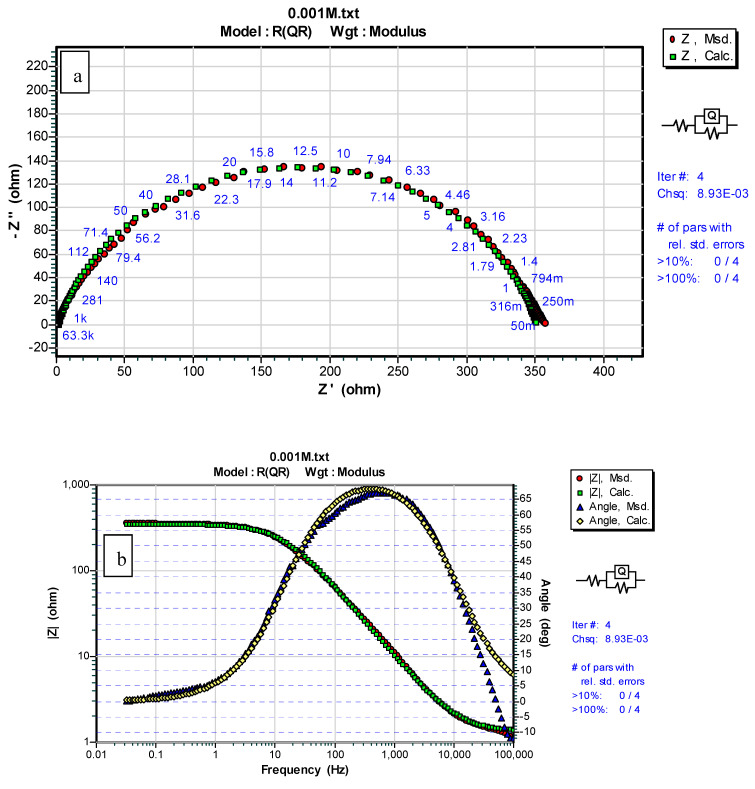
Simulation of EIS (**a**) and Bode (**b**) plots for MS in 1 M HCl in the presence of 0.001 M of CGSES14 at 20 °C.

EIS data were simulated using the same circuit as that shown in Figure 13. A simulation of the EIS and Bode plots made using the ZSimpWin software is shown in Figure 14 and Figure 15. It was observed that the values of Chsq (i.e., a measure of the confidence degree regarding the simulations) ranged from 0.00193 to 0.00893. This indicated the correctness of the proposed equivalent circuit. CPE consists of $Q_{\mathrm{dl}}$ and n (dispersion coefficient), where n determines the inhomogeneous degree of the MS/solution interface resulting from surface roughness, inhibitor adsorption, porous layer formation, etc. $C_{\mathrm{dl}}$ and absolute impedance ($Z_{\mathrm{CPE}}$) can be calculated from the following equation using CPE [41,42]:

(2) $$C_{\mathrm{dl}}=Q_{\mathrm{dl}}\left(\omega_{\max}\right){}^{n-1}$$

(3) $$Z_{\mathrm{CPE}}=Q_{\mathrm{dl}}{}^{-1}\left(i\omega_{\max}\right){}^{-n}$$

where $\omega =2\pi f_{\max}$, $f_{\max}$ is the frequency at the maxim um imaginary element of the impedance. 

The inhibition efficiency (IE) was calculated using the following equation [43,44]:

(4) $$IE=\frac{R{}^{\circ}{}_{\mathrm{ct}}-R{}_{\mathrm{ct}}}{R{}^{\circ}{}_{\mathrm{ct}}}\times 100$$

where $R{}^{\circ}{}_{\mathrm{ct}}$ and $R{}_{\mathrm{ct}}$ are the charge-transfer resistance with and without the inhibitor.

The electrochemical data are summarized in Table 5. It was noted that $R{}_{\mathrm{ct}}$ and $\eta_{I}$ increased with increasing concentrations of CGSES12, CGSES14 and CGSES16. $R{}_{\mathrm{ct}}$ was reached at the maximum concentration (0.0025 M), confirming that the inhibitor prevents the charge transfer of the dissolution and H_2_ evolution processes. $R{}_{\mathrm{ct}}$ was associated with a slower corroding system. $R{}_{\mathrm{ct}}$ and $\eta_{I}$ were reached at the maximum concentration (0.0025 M). The $R{}_{\mathrm{ct}}$ and IE of these materials follow the order:

CGSES16 > CGSES14 > CGSES12

This indicates that the inhibition efficiency increased with increasing the hydrophobic part (fatty alkyl chain) of the tested inhibitors.

The dispersion coefficients (*n*) for CGSES12, CGSES14 and CGSES16 were in the range of 0.75 to 0.98. This means that the MS surface was inhomogeneous, but not the plane. Therefore ideal capacitance ($C_{\mathrm{dl}}$) must be replaced nonideal capacitance (CPE). From Table 2, we noted that $C_{\mathrm{dl}}$ for the inhibited solution was higher than for the uninhibited solution. The decrease in $C_{\mathrm{dl}}$ with increasing the inhibitor concentration was due to the increase in thickness or decrease in the dielectric constant of the adsorption film. This confirmed the adsorption of CGSES12, CGSES14 and CGSES16 molecules on the MS surface, instead of water molecules [45,46].

### 3.4. Weight Loss

The corrosion rate (k) and inhibition efficiency (IE) were calculated using the following equations [47,48]:

(5) $$k=\frac{\Delta W}{At}\;\;$$

(6) $$IE=\frac{\left(k_{\mathrm{free}}-k_{\mathrm{inh}}\right)}{k_{\mathrm{free}}}\times 100$$

where ΔW is the average WL, A is the surface area (cm^2^), t is the time (h), $k_{\mathrm{free}}$ and $k_{\mathrm{inh}}$ the corrosion rates of MS in the blank without and with unlike concentrations of inhibitor, respectively. k and IE of MS were determined in the presence and absence of CGSES12, CGSES14 and CGSES16 in 1 M HCl at dissimilar concentrations and temperatures; see Table 6.

#### 3.4.1. The Effect of Concentration

From Table 6, it can be seen that k decreased while IE increased with increasing the concentrations of CGSES12, CGSES14 and CGSES16 from 0.0001 M to 0.0025 M. This was illustrated by the adsorption mode of CGSES12, CGSES14 and CGSES16 covering the MS surface. At the maximum concentration (0.0025 M), the IE values were 93.93, 96.36 and 97.46% for CGSES12, CGSES14 and CGSES16, respectively, at 20 °C. Thus, these compounds were shown to be effective inhibitors for MS in 1 M HCl. The efficiency of these inhibitors follows the order:

CGSES16 > CGSES14 > CGSES12

#### 3.4.2. Effect of temperature

From Table 6, it can be seen that k increased while IE decreased with increasing the temperature from 20 to 80 °C at all tested concentrations for CGSES12, CGSES14 and CGSES16. This was due to desorption mode of CGSES12, CGSES14 and CGSES16 from the MS surface. The maximum IE value occurred at the maximum concentration (0.0025 M) and at the minimum temperature, i.e., 20 °C, for CGSES12, CGSES14 and CGSES16, respectively. Thus, these compounds may be regarded as effective inhibitors for MS in 1 M HCl. The efficiency of these inhibitors at 20, 40, 60, and 80 °C follows the order:

CGSES16 > CGSES14 > CGSESS12

The inhibition efficiencies obtained from polarization, EIS, and weight loss are compatible.

### 3.5. Standard Adsorption Thermodynamic

CGSES12, CGSES14 and CGSES16 exhibited inhibitive behavior through adsorption on the MS surface.

Therefore, many adsorption isotherms were utilized to fit the experimental data. The Langmuir isotherm was found to be the best model. The Langmuir isotherm is described by the following equation [49,50]:

(7) $$\frac{C}{\theta }=\frac{1}{K_{\mathrm{ads}}}+C$$

where $K_{\mathrm{ads}}$ is the adsorption equilibrium constant, C is the inhibitor concentration and θ is the surface coverage degree.

**Figure 16:**
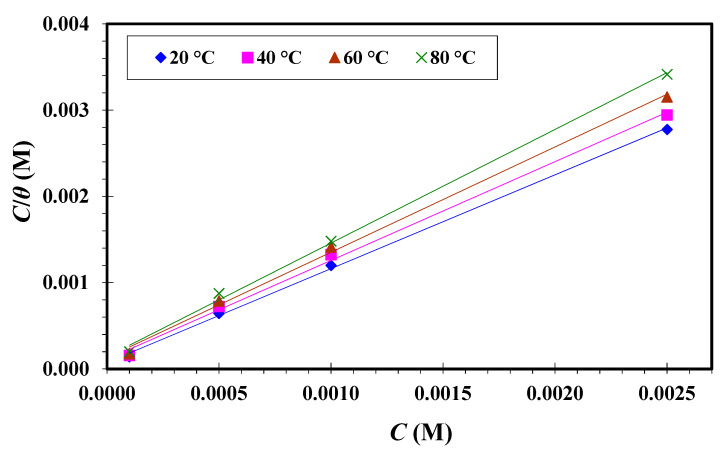
Langmuir isotherm adsorption model for CGSES12 on the MS surface in 1 M HCl at different temperatures.

**Figure 17:**
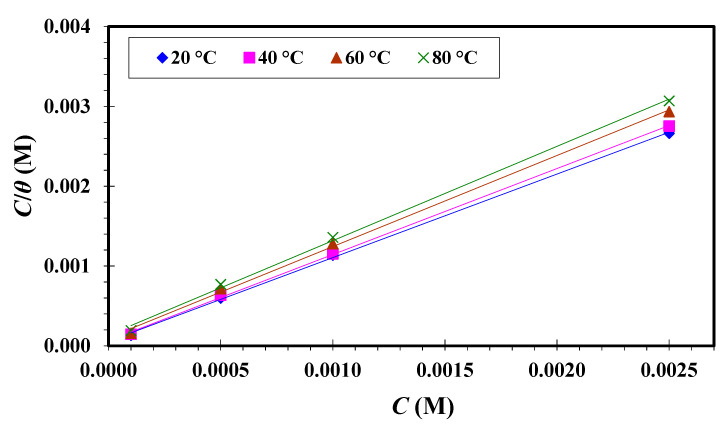
Langmuir isotherm adsorption model for CGSES14 on the MS surface in 1 M HCl at different temperatures.

**Figure 18:**
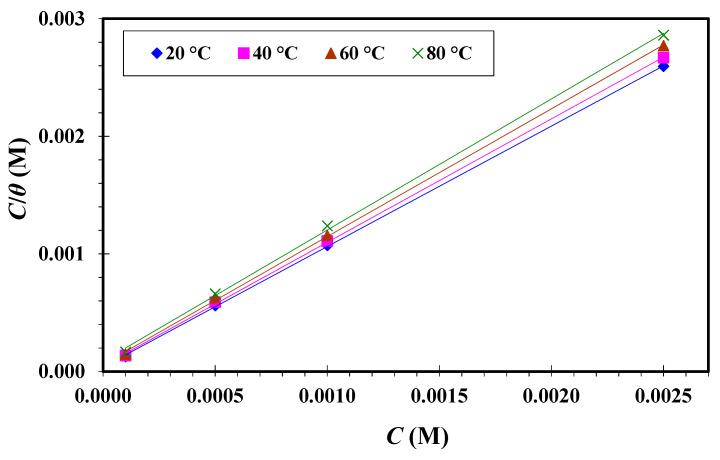
Langmuir isotherm adsorption model for CGSES14 on the carbon steel surface in 1 M HCl at different temperatures.

Linear lines were observed when plotting (C/θ) against C (Figure 16, Figure 17 and Figure 18). The intercept of these lines equaled $\left(1/K_{\mathrm{ads}}\right)$, and the slope and linear correlation coefficients (*R*^2^) were very close to 1. This confirmed that the adsorption of CGSES12, CGSES14 and CGSES16 were consistent with the Langmuir isotherm. The $K_{\mathrm{ads}}$ values are listed in Table 7. Large $K_{\mathrm{ads}}$ values occurred due to strong adsorption of CGSES12, CGSES14 and CGSES16 molecules on the MS surface. The $K_{\mathrm{ads}}$ value increased with increasing the inhibitor concentration. This was due to increasing the numbers of CGSES12, CGSES14 and CGSES16 molecules on the MS surface. The $K_{\mathrm{ads}}$ value decreased with increasing the temperature, which was due to a desorption of CGSES12, CGSES14 and CGSES16 molecules from the MS surface.

The standard adsorption free energy $\left(\Delta G{}^{\circ}{}_{\mathrm{ads}}\right)$ was calculated using Equation (10) [51]:

(8) $$\Delta G{}^{\circ}{}_{\mathrm{ads}}=-RTln\left(55.5K_{\mathrm{ads}}\right)$$

where the value 55.5 is the concentration of water in the test solution expressed in M [52].

The calculated $\Delta G{}^{\circ}{}_{\mathrm{ads}}$ values for CGSES12, CGSES14 and CGSES16 are listed in Table 7. The $\Delta G{}^{\circ}{}_{\mathrm{ads}}$ values were in a range of −33.53 to −39.18 kJ mol^−1^, due to the fact that the adsorption of CGSES12, CGSES14 and CGSES16 was a combination of physical and chemical processes [53,54,55].

$\Delta H{}^{\circ}{}_{\mathrm{ads}}$ was calculated from Van’t Hoff equation [56,57]:

(9) $$\ln K_{{}_{\mathrm{ads}}}=\left(\frac{-\Delta H_{\mathrm{ads}}^{o}}{RT}\right)+\mathrm{constant}$$

**Figure 19:**
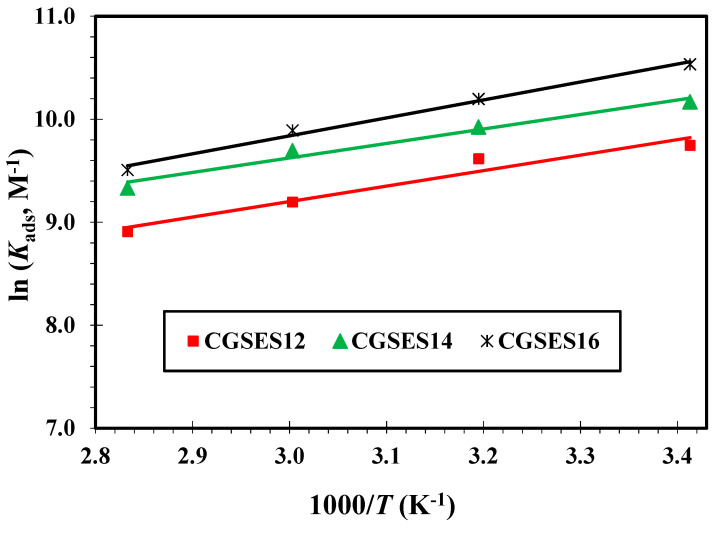
The relationship between ln *K*_ads_ and 1/*T* for MS in a 1 M HCl solution containing different concentrations of the synthesized cationic gemini surfactants on the MS surface in 1 M HCl at different temperatures.

Plotting $\ln K_{\mathrm{ads}}\;\mathrm{vs}.\;\frac{1}{T}$ yielded straight lines, as shown in Figure 19. The slope of the straight line is equal to $\left(\frac{-\Delta H_{\mathrm{ads}}^{o}}{R}\right)$. The negative $\Delta H{}^{\circ}{}_{\mathrm{ads}}$ values indicated that the adsorption of CGSES12, CGSES14 and CGSES16 was an exothermic process.

$\Delta S{}^{\circ}{}_{\mathrm{ads}}$ was calculated from the following equation [58]:

(10) $$\Delta G_{\mathrm{ads}}^{o}=\Delta H_{\mathrm{ads}\;}^{o}-T\Delta S_{\mathrm{ads}}^{o}$$

The positive value of $\Delta S{}^{\circ}{}_{\mathrm{ads}}$ listed in Table 7 indicates an increase in the disorder of CGSES12, CGSES14 and CGSES16 during the adsorption process.

### 3.6. Activation Energy (E_a_)

*E*_a_ was calculated using the Arrhenius equation [59]:

(11) $$lnk=\frac{-E_{a}}{RT}+lnA$$

**Figure 20:**
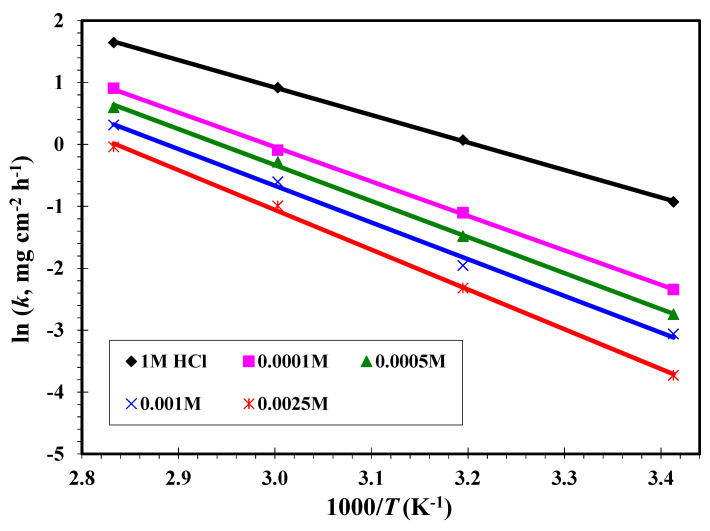
ln *k* versus 1/*T* curves for MS dissolution in the absence and presence of different concentrations of CGSES12 in 1 M HCl solution.

**Figure 21:**
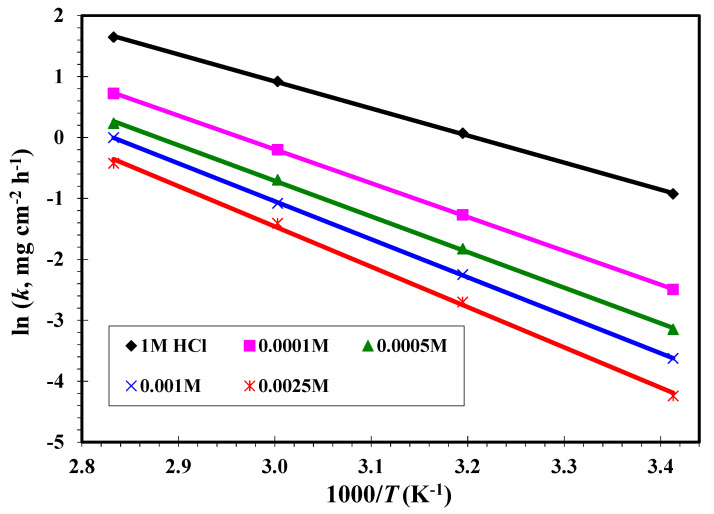
ln *k* versus 1/*T* curves for MS dissolution in the absence and presence of different concentrations of CGSES14 in 1 M HCl solution.

**Figure 22:**
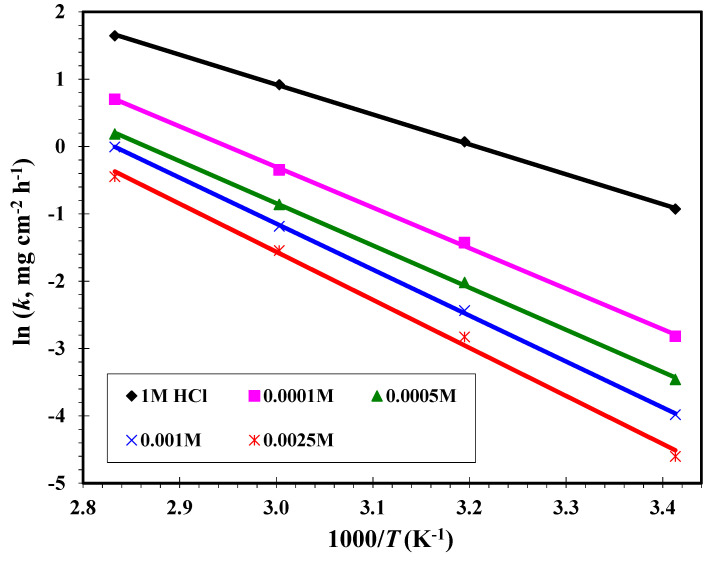
ln *k* versus 1/*T* curves for MS dissolution in the absence and presence of different concentrations of CGSES16 in 1 M HCl solution.

A plot of lnk vs. (1/T) yielded straight lines, as shown in Figure 20, Figure 21 and Figure 22. The slope of the straight line equaled $\left(-E_{a}/R\right)$. The $E_{a}$ values were calculated and are listed in Table 8. The $E_{a}$ values in the presence of CGSES12, CGSES14 and CGSES16 were higher than in their absence. This confirmed the adsorption of CGSES12, CGSES14 and CGSES16 on the MS surface in 1 M HCl [60].

### 3.7. Surface Active Properties

#### 3.7.1. Critical Micelle Concentration $\left(C_{\mathrm{cmc}}\right)$ and Effectiveness $\left(\pi_{\mathrm{cmc}}\right)$

**Figure 23:**
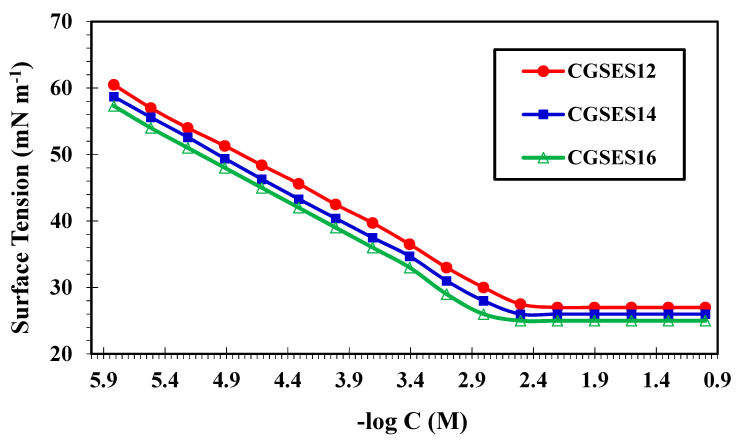
Variation of the surface tension with the concentration of the synthesized cationic gemini surfactant in double-distilled water at 25 °C.

The surface tension (γ) vs. (logC), as signified in Figure 23, was drawn to determine the $C_{\mathrm{cmc}}$ value. $C_{\mathrm{cmc}}$ was determined from the break point of the plots, as listed in Table 9. It was observed that CGSES12, CGSES14 and CGSES16 strongly reduced the surface tension. The effectiveness $\left(\pi_{\mathrm{cmc}}\right)$ at $C_{\mathrm{cmc}}$, is estimated from the following equation [61,62]:

(12) $$\pi_{\mathrm{cmc}}=\gamma_{o}-\gamma_{\mathrm{cmc}}$$

where $\gamma_{o}$ and $\gamma_{\mathrm{cmc}}$ are the surface tension of pure water and at $C_{\mathrm{cmc}}$.

The values of $\gamma_{\mathrm{cmc}}$ and $\pi_{\mathrm{cmc}}$ for CGSES12, CGSES14 and CGSES16 are given in Table 9. It was found that $C_{\mathrm{cmc}}$ decreased while $\pi_{\mathrm{cmc}}$ increased with increasing the hydrophobic chain.

#### 3.7.2. Surface Excess $\left(\Gamma_{\max}\right)$ and Minimum Surface Area $\left(A_{\min}\right)$

$\Gamma_{\max}$ and $A_{\min}$ are the concentration and minimum area of the surfactant at the air–solution interface. $\Gamma_{\max}$ and $A_{\min}$ were computed using Gibbs’s adsorption [63,64]:

(13) $$\Gamma_{\max}=\left(\frac{-1}{nRT}\right)\left(\frac{d\gamma }{d\ln C}\right)$$

(14) $$A_{\min}=\frac{10^{14}}{N_{A}\Gamma_{\max}}$$

By plotting γ against lnC, the slope refers to dγ/dlnC. The surfactant concentration at the surface was always higher than that in the bulk solution [65,66,67].

The $\Gamma_{\max}$ and $A_{\min}$ values were calculated and are shown in Table 9. It was found that the $\Gamma_{\max}$ value increased while $A_{\min}$ decreased with increasing the hydrophobic chain. These results indicate that CGSES12, CGSES14 and CGSES16 are good surfactants.

### 3.8. Comparison between the synthseized surfactants and other inhibitors reported in the literature (Correlation Structure and Efficiency-Surface Activity)

The effectiveness of the synthesized inhibitors is dependent on their chemical structure and surface properties. CGSES12, CGSES14 and CGSES16, in addition to the fatty alkyl chain, were adsorbed on the MS surface by a lone pairs of electrons of N and O atoms, N^+^ and Cl^–^ ions. The $\Delta G{}^{\circ}{}_{\mathrm{ads}}$ values of CGSES12, CGSES14 and CGSES16 were −33.53, −34.55 and −35.44 kJ mol^–1^ at 20 °C, and gradually declined to −37.93, −39.18 and −39.69 kJ mol^–1^ at 80 °C, respectively. Furthermore, the $\Delta H{}^{\circ}{}_{\mathrm{ads}}$ values of CGSES12, CGSES14 and CGSES16 were negative and low (−12.50, −11.70 and −14.49 kJ mol^–1^). Such values suggest that depending on the temperature value, the adsorption of CGSES12, CGSES14 and CGSES16 was physical and chemical in nature. The physical adsorption between the charged MS surface and the charged atoms (N^+^ and Cl^–^) of CGSES12, CGSES14 and CGSES16 occurred at low temperatures. By increasing the temperature, the chemical adsorption of CGSES12, CGSES14 and CGSES16 arose due to donor–acceptor interactions between the free electron pairs of the hetero atoms (O and N) and vacant d-orbitals of iron.

**Figure 24:**
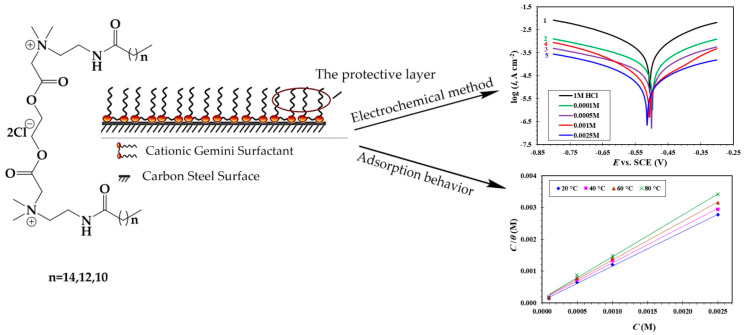
Dependence of the efficiency of the synthesized inhibitors on the chemical structure and surface properties.

The corrosion inhibition efficiency (IE) and reduction of surface tension (effectiveness, $\pi_{\mathrm{cmc}}$) of these inhibitors follows the order CGSES16 > CGSES14 > CGSES12. The adsorption of these cationic gemini surfactants resulted in well packed, adsorbed layers, a greater covered surface area and more homogeneous adsorbed film, as shown in Figure 24.

Surfactants are very effective corrosion inhibitors which lower the surface tension (or interfacial tension) between the corrosive medium and the MS surface. The evaluation of surfactants as corrosion inhibitors usually uses concentrations below critical micelle concentration ($C_{\mathrm{cmc}}$) because the surfactants molecules after $C_{\mathrm{cmc}}$ form micelle in the majority of tested solutions (a distinctive property of surfactants). Therefore, any concentration greater than $C_{\mathrm{cmc}}$ will not increase the inhibition efficiency. On the other hand, organic compounds disperse almost at the same rate, both in bulk solution and at the interface. A comparison between the tested compounds and other inhibitors reported in the literature for anticorrosion protection of steel under the same experimental conditions is summarized in Table 10. This comparison shows that synthesized cationic gemini surfactants were more effective as corrosion inhibitors compared with other surfactants.

## 4. Conclusions

The present work concludes with the following points:

1. Three new cationic gemini surfactants with ester spacer type of 2-2′-(ethane-1,2-diyl bis(oxy)) bis(N-(2-alkanamidoethyl)-N,N-dimethyl-2-oxoethan-1-aminium)) dichloride) (CGSES12, CGSES14, and CGSES16) based on N,N dimethyl fatty amido ethylamine were synthesized using a three-step reaction method, and were characterized by FT-IR, ^1^HNMR, ^13^CNMR spectroscopies.
2. The inhibition performance of the three surfactants was studied by weight loss and electrochemical measurements.
3. The results showed that CGSES12, CGSES14 and CGSES16 behaved as effective inhibitors and surface agents.
4. The maximum efficiency was higher than 94% at 2.5 mM, and the inhibition order was CGSES16 > CGSES14 > CGSES12. This was due to the increment in hydrophobicity of the gemini surfactants.
5. Their adsorption on the MS surface followed the Langmuir isotherm. CGSES12, CGSES14 and CGSES16 can be considered mixed type inhibitors.
6. The presence of CGSES12, CGSES14 and CGSES16 increased the charge transfer resistance and decreased the corrosion rate. The adsorption focused on the heteroatoms and the surface properties of the cationic gemini surfactants.
7. $\pi_{\mathrm{cmc}}$ and $\Gamma_{\max}$ increased, while $C_{\mathrm{cmc}}$ and $A_{\min}$ decreased with increasing the hydrophobic chain.

## Figures and Tables

**Figure 3:**
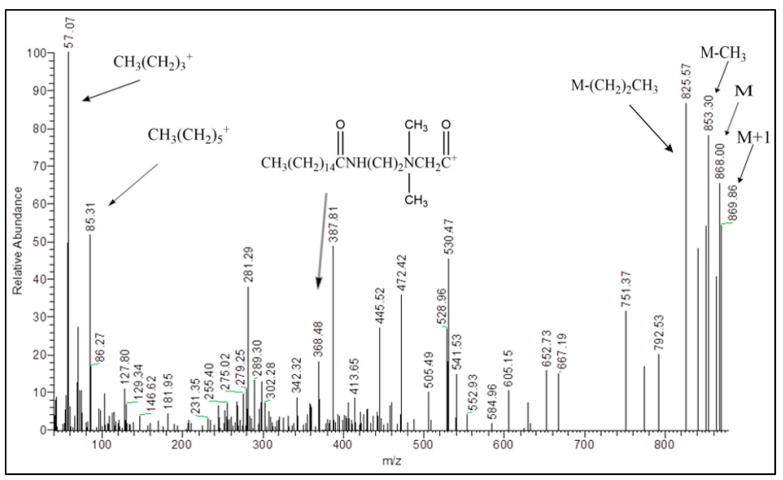
Mass spectrum for CGSES16.

**Table 1:** FT-IR data of cationic gemini surfactant.

Cationic Gemini Surfactants	FT-IR Data (cm^−1^)				
	(ν N–H)	ν C-H Asym.	ν C-H Sym	ν C=O Ester	ν C=O Amide
CGSES12	3329	2925	2842	1740	1641
CGESE14	3324	2919	2842	1747	1641
CGSES16	3352	2911	2850	1755	1648

**Table 2:** ^1^H NMR reading describing the type of proton in CGSES12-16.

Cationic Gemini Surfactants	Proton NMR (δ in ppm, 850 MHz, CDCl_3_)									
	Tail				Amide	Amido-Amine			Spacer	
	CH_3_ (a)	CH_2_ (b)	CH_2_ (c)	CH_2_ (d)	NH (e)	CH_2_ (f)	CH_2_ (g)	CH_3_ (h)	CH_2_ (i)	CH_2_ (j)
CGSES12	0.83	1.16	1.51	2.16	8.42	3.89	3.74	3.51	4.45	5.03
CGESE14	0.86	1.20	1.55	2.19	8.45	3.92	3.72	3.57	4.49	5.01
CGSES16	0.86	1.23	1.58	2.28	8.48	3.67	3.57	3.53	4.48	5.08

**Table 3:** GC-MS data of CGSES12-16.

Cationic Gemini Surfactants	Base Peak	Proposed Structure
CGSES12	502.45	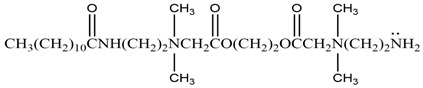
CGSES14	470.48	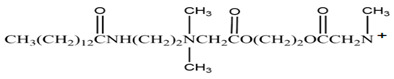
CGSES16	57.07	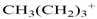

**Table 4:** Potentiodynamic polarization parameters for CS in 1 M HCl with and without different concentrations of the synthesized cationic gemini surfactants at 20 °C.

Inhibitor Name	Conc. of Inhibitor(M)	*E*_corr_ (mV vs. SCE)	*i*_corr_ (mA cm^−2^)	*β*_a_ (mV dec^−1^)	*β*_c_ (mV dec^−1^)	*IE* (%)
**-**	0.00	−508.4	0.4134	135.8	−162.5	—
**CGSES12**	1.0 × 10^−4^	−510.9	0.1041	160.0	−181.3	74.82
	5.0 × 10^−4^	−513.5	0.0665	162.5	−183.3	83.91
	1.0 × 10^−3^	−514.8	0.0481	125.4	−147.1	88.36
	2.5 × 10^−3^	−516.1	0.0325	165.9	−187.3	92.14
**CGSES14**	1.0 × 10^−4^	−509.8	0.0877	114.6	−192.9	78.79
	5.0 × 10^−4^	−510.5	0.0467	161.8	−174.6	88.70
	1.0 × 10^−3^	−508.3	0.0281	163.4	−177.1	93.20
	2.5 × 10^−3^	−513.2	0.0175	182.5	−178.3	95.77
**CGSES16**	1.0 × 10^−4^	−507.8	0.0677	171.7	−157.8	83.62
	5.0 × 10^−4^	−509.5	0.0356	155.9	−190.1	91.39
	1.0 × 10^−3^	−517.8	0.0196	184.2	−182.0	95.26
	2.5 × 10^−3^	−513.8	0.0149	185.5	−195.3	96.40

**Table 5:** Electrochemical impedance parameters for CS in 1 M HCl with and without different concentrations of the synthesized cationic gemini surfactants at 20 °C.

Inhibitor Name	Conc. of Inhibitor (M)	*R*_s_ (Ω cm^2^)	*Q*_dl_ (mΩ^−1^ s^n^ cm^−2^)	*n*	*R*_ct_ (Ω cm^2^)	*Chsq*	*C*_dl_ (μF cm^−2^)	*IE* (%)
**-**	0.00	1.80	0.7691	0.83	24.73	0.00506	97.8	-
**CGSES12**	1.0 × 10^−4^	2.39	0.2192	0.82	96.45	0.00427	28.3	74.36
	5.0 × 10^−4^	1.75	0.1385	0.82	152.8	0.00654	18.6	83.82
	1.0 × 10^−3^	1.78	0.1097	0.92	199.6	0.00665	14.3	87.61
	2.5 × 10^−3^	1.92	0.0674	0.79	362.8	0.00848	8.1	93.18
**CGSES14**	1.0 × 10^−4^	1.50	0.1848	0.75	120.7	0.00426	24.5	79.51
	5.0 × 10^−4^	1.30	0.1032	0.87	211.0	0.00764	13.8	88.28
	1.0 × 10^−3^	1.33	0.0637	0.83	349.8	0.00893	9.3	92.93
	2.5 × 10^−3^	1.64	0.0364	0.77	573.5	0.00281	5.1	95.69
**CGSES16**	1.0 × 10^−4^	1.44	0.1377	0.75	162.4	0.00785	17.8	84.77
	5.0 × 10^−4^	1.93	0.0792	0.74	280.5	0.00704	9.9	91.18
	1.0 × 10^−3^	1.79	0.0411	0.96	502.9	0.00237	5.7	95.08
	2.5 × 10^−3^	1.66	0.0327	0.88	671.1	0.00193	4.2	96.32

**Table 6:** Weight loss results for MS in 1 M HCl in the absence and presence of different concentrations of the synthesized cationic gemini surfactants at various temperatures.

Inhibitor Name	Inhibitor conc. (M)	20 °C			40 °C			60 °C			80 °C		
		*k* (mg cm^−^^2^ h^−^^1^)	*Ө*	*IE* (%)	*k* (mg cm^−^^2^ h^−^^1^)	*Ө*	*IE* (%)	*k* (mg cm^−^^2^ h^−^^1^)	*Ө*	*IE* (%)	*k* (mg cm^−^^2^ h^−^^1^)	*Ө*	*IE* (%)
Absence	1 M HCl	0.3954	—	—	1.0712	—	—	2.5040	—	—	5.1868	—	—
CGSES12	1.0 × 10 ^−4^	0.0960	0.76	75.73	0.3318	0.69	69.03	0.9095	0.64	63.68	2.4744	0.52	52.29
	5.0 × 10 ^−4^	0.0643	0.84	83.73	0.2269	0.79	78.81	0.7539	0.70	69.89	1.8189	0.65	64.93
	1.0 × 10 ^−3^	0.0469	0.88	88.14	0.1418	0.87	86.76	0.5465	0.78	78.17	1.3691	0.74	73.60
	2.5 × 10 ^−3^	0.0240	0.94	93.93	0.0983	0.91	90.82	0.3709	0.85	85.19	0.9605	0.81	81.48
CGSES14	1.0 × 10 ^−4^	0.0825	0.79	79.14	0.8164	0.67	67.40	0.8164	0.67	67.40	2.0565	0.60	60.35
	5.0 × 10 ^−4^	0.0429	0.89	89.14	0.2806	0.74	73.81	0.4985	0.80	80.09	1.2630	0.76	75.65
	1.0 × 10 ^−3^	0.0266	0.93	93.27	0.1606	0.85	85.01	0.3392	0.86	86.45	0.9983	0.81	80.75
	2.5 × 10 ^−3^	0.0144	0.96	96.36	0.1053	0.90	90.17	0.2444	0.90	90.24	0.6530	0.87	87.41
CGSES16	1.0 × 10 ^−4^	0.0597	0.85	84.90	0.2398	0.78	77.62	0.7063	0.72	71.79	2.0154	0.64	64.38
	5.0 × 10 ^−4^	0.0314	0.92	92.05	0.1327	0.88	87.61	0.4231	0.83	83.10	1.2015	0.79	78.77
	1.0 × 10 ^−3^	0.0186	0.95	95.29	0.0874	0.92	91.84	0.3054	0.88	87.80	0.9931	0.82	82.45
	2.5 × 10 ^−3^	0.0100	0.97	97.46	0.0591	0.94	94.48	0.2133	0.91	91.48	0.6388	0.89	88.71

**Table 7:** Standard thermodynamic parameters of adsorption on the MS surface in 1 M HCl containing different concentrations of the synthesized cationic gemini surfactants.

Inhibitor	Temperature (°C)	*K*_ads_ (M^−1^)	Δ*G*°_ads_ (kJ mol^−1^)	Δ*H*°_ads_ (kJ mol^−1^)	Δ*S*°_ads_ (J mol^−1^ K^−1^)
CGSES12	20	17,097	−33.53	−12.50	71.77
	40	15,022	−35.48		73.42
	60	9858	−36.58		72.31
	80	7398	−37.93		72.05
CGSES14	20	26,074	−34.55	−11.70	78.02
	40	20,439	−36.28		78.55
	60	16,225	−37.96		78.87
	80	11,295	−39.18		77.85
CGSES16	20	37,549	−35.44	−14.49	71.51
	40	26,806	−36.99		71.87
	60	19,746	−38.50		72.11
	80	13,460	−39.69		71.39

**Table 8:** Activation energy (*E*_a_, kJ mol^−1^) values for MS in 1 M HCl in the absence and presence of different concentrations of the synthesized cationic gemini surfactants.

Conc. of Inhibitor (M)	*E*_a_ (kJ mol^−1^)		
	CGSES12	CGSES14	CGSES16
**1 M HCl**	36.91	36.91	36.91
**1.0 × 10^−4^**	46.28	46.13	50.11
**5.0 × 10^−4^**	48.36	48.59	52.07
**1.0 × 10^−3^**	49.31	51.82	56.74
**2.5 × 10^−3^**	53.41	54.95	59.32

**Table 9:** Surface tension parameters of the synthesized cationic gemini surfactants in double-distilled water at 25 °C.

Inhibitor name	*C*_cmc_ x 10^3^ (M)	*γ*_cmc_ (mN m^−^^1^)	*π*_cmc_ (mN m^−^^1^)	*Γ*_max_ × 10^11^ (mol cm^−^^2^)	*A*_min_ (nm^2^)
**CGSES12**	2.60	27.0	45.0	5.82	2.85
**CGSES14**	1.70	26.2	45.8	5.92	2.81
**CGSES16**	1.10	25.4	46.6	6.01	2.76

**Table 10:** The comparison between the tested compounds and other inhibitors reported in the literature for anti-corrosion protection of steel at the same experiment conditions.

Inhibitor Name	IE (%)	Reference
(E)-decyl-4-[(2-hydroxyethylamino) methyl]-N,N dimethyl benzenaminium bromide	90.26	[68]
(E)-dodecyl-4-[(2-hydro-xyethylamino)methyl]-N,N dimethyl benzenaminium bromide	90.95	[68]
(E)-hexadecyl4-[(2-hydro-xyethylamino)methyl]-N,N-dimethyl benzenaminium bromide	93.12	[68]
N,N-bis(2-hydroxyethyl)-N-octylbenzo[d]thiazol 2-amonium bromide	87.26	[69]
N,N-bis(2-hydroxyethyl)-N-decylbenzo[d]thiazol 2-amonium bromide	91.46	[69]
N,N-bis(2-hydroxyethyl)-Ndodecylbenzo[d] thiazol-2 -amonium bromide	94.35	[69]
N-(3-(benzylideneamino)propyl)-N,N-dimethyldecan-1-ammonium bromide	82.93	[70]
N-(3-(benzylideneamino)propyl)-N,N-dimethyldodecan-1-ammonium bromide	87.45	[70]
N-(3-(benzylideneamino)propyl)-N,N-dimethylhexadecan-1-ammonium bromide	88.02	[70]
2-2′-(Ethane-1,2-diyl bis(oxy)) bis(N-(2-dodecanamidoethyl)-N,N-dimethyl-2-oxoethan-1-aminium)) dichloride (CGSES12)	93.93	This work
2-2′-(Ethane-1,2-diyl bis(oxy)) bis(N-(2-tetradecanamidoethyl)-N,N-dimethyl-2-oxoethan-1-aminium)) dichloride (CGSES14)	96.36	This work
2-2′-(Ethane-1,2-diyl bis(oxy)) bis(N-(2-hexadecanamidoethyl)-N,N-dimethyl-2-oxoethan-1-aminium)) dichloride (CGSES16)	97.46	This work

## Supplementary Materials

The following are available online at https://www.mdpi.com/1996-1944/13/12/2790/s1, Figure S1. FT-IR Spectra N-(2-(dimethylamino)ethyl)dodecanamide, Figure S2. FT-IR Spectra for N-(2-(dimethylamino)ethyl)tetradecanamide, Figure S3. FT-IR Spectra for N-(2-(dimethylamino)ethyl) hexadecanamide*,* Figure S4. FT-IR Spectra for Ethane-1,2-diyl bis(2-chloroethanoat), Figure S5. FT-IR Spectra for (2-2’-(ethane-1,2-diylbis(oxy))bis(N-(2-dodecanamidoethyl)-N,N-dimethyl-2-oxoethan-1-aminium)) dichloride, Figure S6. FT-IR Spectra for 2-2’-(ethane-1,2-diylbis(oxy))bis(N-(2-hexadecanamidoethy)-N,N-dimethyl-2-oxoethan-1-aminium) dichloride, Figure S7. ^1^H-NMR Spectra for N-(2-(dimethylamino)ethyl)dodecanamide*,* Figure S8. ^1^H-NMR Spectra for N-(2-(dimethylamino)ethyl)tetradecanamid, Figure S9. ^1^H-NMR Spectra for N-(2-(dimethylamino)ethyl) hexadecanamide), Figure S10. ^1^H-NMR Spectra for Ethane-1,2-diyl bis(2-chloroethanoat), Figure S11.^1^H-NMR Spectra for (2-2’-(ethane-1,2-diylbis(oxy))bis(N-(2-dodecanamidoethyl)-N,N-dimethyl-2-oxoethan-1-aminium)) dichloride, Figure S12. ^13^C-NMR Spectra for. N-(2-(dimethylamino)ethyl) hexadecanamide, Figure S13. Mass spectra for2-2’-(ethane-1,2-diylbis(oxy))bis(N-(2-dodecanamidoethyl)-N,N-dimethyl-2-oxoethan-1-aminium)) dichloride, Figure S14 Mass spectra for 2,2’-(ethane-1,2-diylbis(oxy))bis(N,N-dimethyl-2-oxo-N-(2-tetradecanamidoethyl)ethan-1-aminium)di chloride.
