# Supplementary material for: Adsorption and Corrosion Performance of New Cationic Gemini Surfactants Derivatives of Fatty Amido Ethyl Aminium Chloride with Ester Spacer for Mild Steel in Acidic Solutions
Source: Materials (Basel). 2020 Jun 20;13(12):2790. doi: 10.3390/ma13122790 (PMC7345868; doi:10.3390/ma13122790)
Supplement: Supplementary file 1 [file materials-13-02790-s001.pdf]

Supplementary Materials

S2.1 Materials

Dodecanoyl, tetradecanoyl, octadecanoyl chloride and *N,N'*-dimethyl ethylenediamine were purchased from Sigma-Aldrich (USA). Hexadecanoyl chloride and ethylene glycol purchased from across organics. 2- chloroacetyl chloride was bought from Loba-Chemie (India).All other solvents were reagent grade. Water used for experiments was deionized water.

S2.2. Structural characterization

Characterizations of cationic Gemini surfactants (CGSES12-16) were performed with the assistance of FT-IR, ^{1}H-NMR and ^{13}C-NMR MALDI MS techniques. Using a Tescan Shimadzu, Model 8000, Japan, Fourier transform infrared (FT-IR) spectra were obtained within the range 4000–400 cm^{-1}. 1H-NMR spectra are recorded at 20°C on a Varian VXR-300 multinuclear pulsed NMR spectrometer operating at the 1H resonance frequency of 850 MHz experiments. With TMS as an internal standard, the samples were dissolved in $CDCl_3$. Mass spectral analysis was performed with an Agilent (Santa Clara, CA, USA) 6310 ESI–ion trap mass spectrometer.

S2.3. Surface tension technique

The surface tension of aqueous solutions for the surfactant was measured at 25 °C using the Du Nouy ring method with a Kruss K6 tensiometer. The surface properties of the surfactant were evaluated using surface activity parameters, such as the critical micelle concentration (C_{cmc}), surface tension at C_{cmc} (γ_{cmc}), maximum surface excess concentration (Γ_{max}), effectiveness (π_{cmc}), and minimum surface area per molecule (A_{min}) at the air/solution interface.

3.1. Construction Explanation

N-(2-(dimethylamino)ethyl)dodecanamide (DAEA12)

White powder (79% yield); mp 46-49ºC. FTIR (ν in cm^{-1}) 3285 (ν N–H), 1643 (νC=O amide).^{1}H NMR (δ in ppm, CDCl3 solvent at 850 MHz): 0.88 (C**H**$_3$, t), 1.26 ((C**H**$_2$)$_8$, s), 1.62 ($CH_3(CH_2)_8C$**H**$_2CH_2CO$, h), 2.18 (C**H**$_2$-CO-NH, t)), 2.30 (N-(C**H**$_3$)$_2$, s), 2.48 (NH-C**H**$_2$-CH_2, t), 3.36 (CO-NH-C**H**$_2$, q), 6.22 (N**H**, s).

N-(2-(dimethylamino)ethyl)tetradecanamide (DAEA14)

White powder (81% yield); mp 48-50ºC. FTIR (ν in cm^{-1}) 3024 (ν N–H), 1634 (νC=O amide).^{1}H NMR (δ in ppm, CDCl3 solvent at 850 MHz): 0.88 (C**H**$_3$, t), 1.26 ((C**H**$_2$)$_{10}$, s), 1.61 ($CH_3(CH_2)_{10}C$**H**$_2CH_2CO$, h), 2.18 (C**H**$_2$-CO-NH, t), 2.29 (N-(C**H**$_3$)$_2$, s), 2.47 (NH-C**H**$_2CH_2$, t), 3.35 (CO-NH-C**H**$_2$, q), 6.20 (N**H**, s).

N-(2-(dimethylamino)ethyl)hexadecanamide (DAEA16)

White crystals (95% yield); mp 64-65ºC. FTIR (ν in cm^{-1}) 3285 (ν N–H), 1634 (νC=O amide).^{1}H NMR (δ in ppm, CDCl3 solvent at 850 MHz): 0.85 (C**H**$_3$, t), 1.23 ((C**H**$_2$)$_{12}$, s), 1.58 ($CH_3(CH_2)_{12}C$**H**$_2CH_2CO$, h), 2.15 (C**H**$_2$-CO-NH, t), 2.25 (N-(C**H**$_3$)$_2$, s), 2.43 (NH-C**H**$_2CH_2$, t), 3.32 (CO-NH-C**H**$_2$, q), 6.21 (N**H**, s). ^{13}C NMR (δ in ppm, CDCl3 solvent at 213 MHz):14.15, 22.71, 25.81, 29.35, 29.40, 29.54, 29.67, 31.94, 36.49, 36.76, 45.03, 57.93, 175.

ES, Colorless columnar crystal (75.1% yield). FTIR (ν in cm^{-1}) 2797 (ν C-H), 1847 (νC=O ester).^{1}H NMR (δ in ppm, CDCl3 solvent at 850 MHz): 4.13 (-O-CH2×2, s), 4.41 (O=C-CH2 ×2, s).

2-2'-(Ethane-1,2-diyl bis(oxy)) bis(N-(2-dodecanamidoethyl)-N,N-dimethyl-2-oxoethan-1-aminium)) dichloride (**CGSES12**)

CGSES12: White wax (85% yield); mp 168-170ºC.FTIR (ν in cm^{-1}): 3304 (ν N–H), 2933 (νC-H asymmetric), 2836 (νC-H symmetric), 1761 (ester), 1634 (amide).^{1}H NMR (δ in ppm, CDCl3 solvent at 850 MHz): 0.83 (C**H**$_3$ × 2, t), 1.16 ((C**H**$_2$)$_8$ × 2, m), 1.51

$(CH_3(CH_2)_8C\underline{H}_2 \times 2$, m), 2.16 ($C\underline{H}_2$-CO-NH × 2, m), 3.51 ($C\underline{H}_3$ × 4, s), 3.79 (N-$C\underline{H}_2$ × 2, m), 3.89 (CO-NH-$C\underline{H}_2$ × 2, m), 4.45 (N-$C\underline{H}_2$-CO × 2, m), 5.03 ($C\underline{H}_2$-O × 2 , m), 8.42 (N**H** × 2 , s). MS m/z (parent ions): 756.58 (M+1).

2-2'-(ethane-1,2-diyl bis(oxy)) bis(N-(2-tetradecanamidoethyl)-N,N-dimethyl-2-oxoethan-1-aminium)) dichloride (**CGSES14)**

Milky wax (81.4 % yield); mp 177-180ºC. FTIR (ν in cm^{-1}): 3324 (ν N–H), 2923 (νC-H asymmetric), 2836 (νC-H symmetric), 1741 (ester), 1634 (amide) .^{1}H NMR (δ in ppm, CDCl3 solvent at 850 MHz): 0.86 ($C\underline{H}_3$ × 2, t), 1.20 (($C\underline{H}_2)_{10}$ × 2, m), 1.55 ($CH_3(CH_2)_{10}C\underline{H}_2$ × 2, m), 2.19 ($C\underline{H}_2$-CO-NH × 2, m), 3.57 ($C\underline{H}_3$ × 4, s), 3.72 (N-$C\underline{H}_2$ × 2, m), 3.92 (CO-NH-$C\underline{H}_2$ × 2, m), 4.49 (N-$C\underline{H}_2$-CO × 2, m), 5.01 ($C\underline{H}_2$-O ×2 , m), 8.45 (N**H** × 2 , s). MS m/z (parent ions): 812.84 (M).

2-2'-(ethane-1,2-diyl bis(oxy)) bis(N-(2-hexadecanamidoethyl)-N,N-dimethyl-2-oxoethan-1-aminium)) dichloride (**CGSES16)**

White crystal (90 % yield); mp 187-188ºC.FTIR (ν in cm^{-1}): 3314 (ν N–H), 2904 (νC-H asymmetric), 2836 (νC-H symmetric), 1751 (ester), 1643 (amide).^{1}H NMR (δ in ppm, CDCl3 solvent at 850 MHz): 0.86 ($C\underline{H}_3$ × 2, t), 1.23 (($C\underline{H}_2)_{12}$ × 2, m), 1.58 ($CH_3(CH_2)_{12}C\underline{H}_2$ × 2, m), 2.28 ($C\underline{H}_2$-CO-NH × 2, m), 2.48 ($C\underline{H}_3$ × 4, s), 3.75 (N-$C\underline{H}_2$× 2, m), 3.96 (CO-NH-$C\underline{H}_2$ × 2, m), 4.52 (N-$C\underline{H}_2$-CO × 2, m), 5.08 ($C\underline{H}_2$-O ×2 , m), 8.48 (N**H** × 2 , s). MS m/z (parent ions): 869.96 (M+1).

Table S1 FT-IR data of DAEA12-16.

DAEA	FT-IR Data(cm^{-1})			
	(ν N–H)	ν C-H Asym.	ν C-H Sym.	**ν C=O Amide**
DAEA12	3291	2911	2834	**1634**
DAEA14	3291	2911	2842	**1634**
DAEA16	3298	2919	2842	**1643**

Table S2 1H NMR reading describing the type of proton in DAEA 12-16

DAEA	Proton NMR (δ in ppm, 850 MHz, $CDCl_3$)							
	CH_3 (a)	CH_2 (b)	CH_2 (c)	CH_2 (d)	NH (e)	CH_2 (f)	CH_2 (g)	**CH_3 (h)**
DAEA12	0.88	1.26	1.62	2.18	6.22	3.36	2.48	**2.30**
DAEA14	0.88	1.26	1.61	2.18	6.20	3.35	2.47	**2.29**
DAEA16	0.85	1.23	1.58	2.15	6.21	3.32	2.43	**2.25**

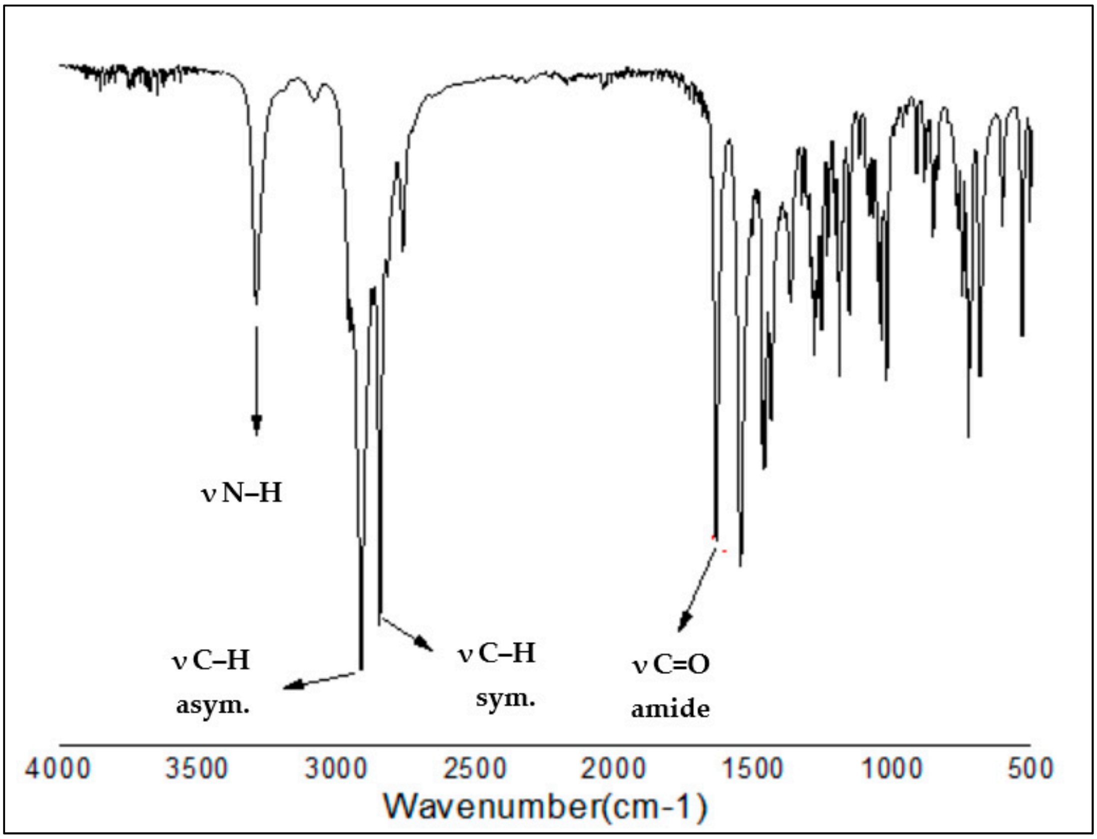

Figure S1 FT-IR Spectra for DAEA12.

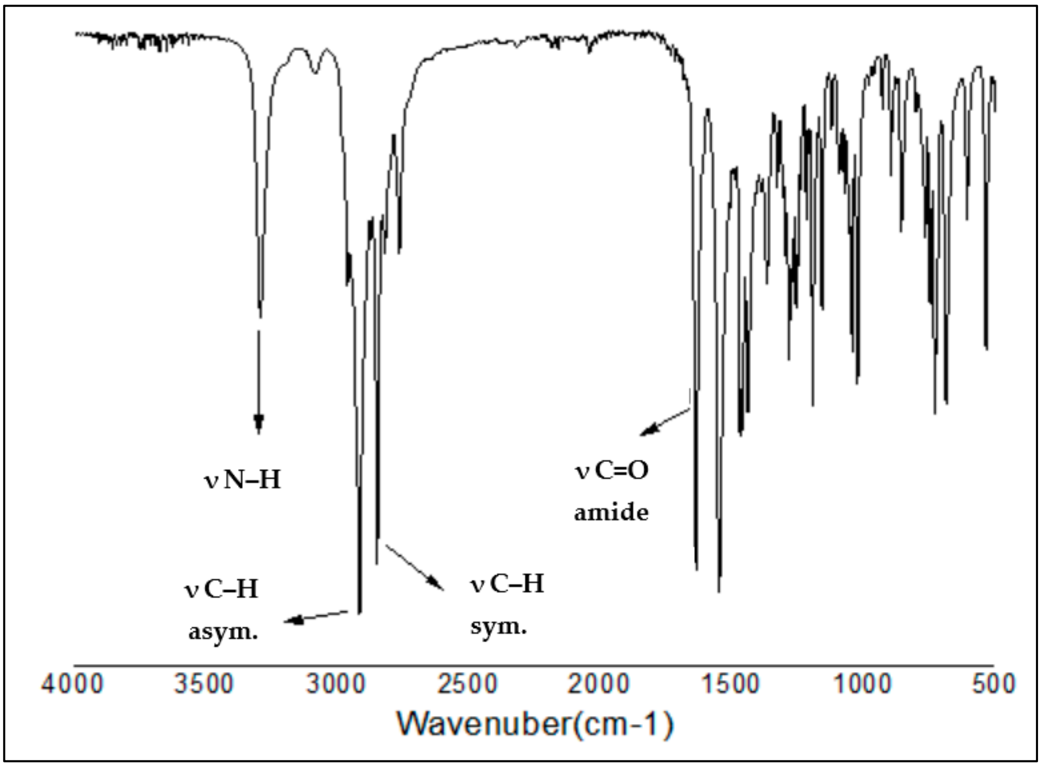

Figure S2 FT-IR Spectra for DAEA14.

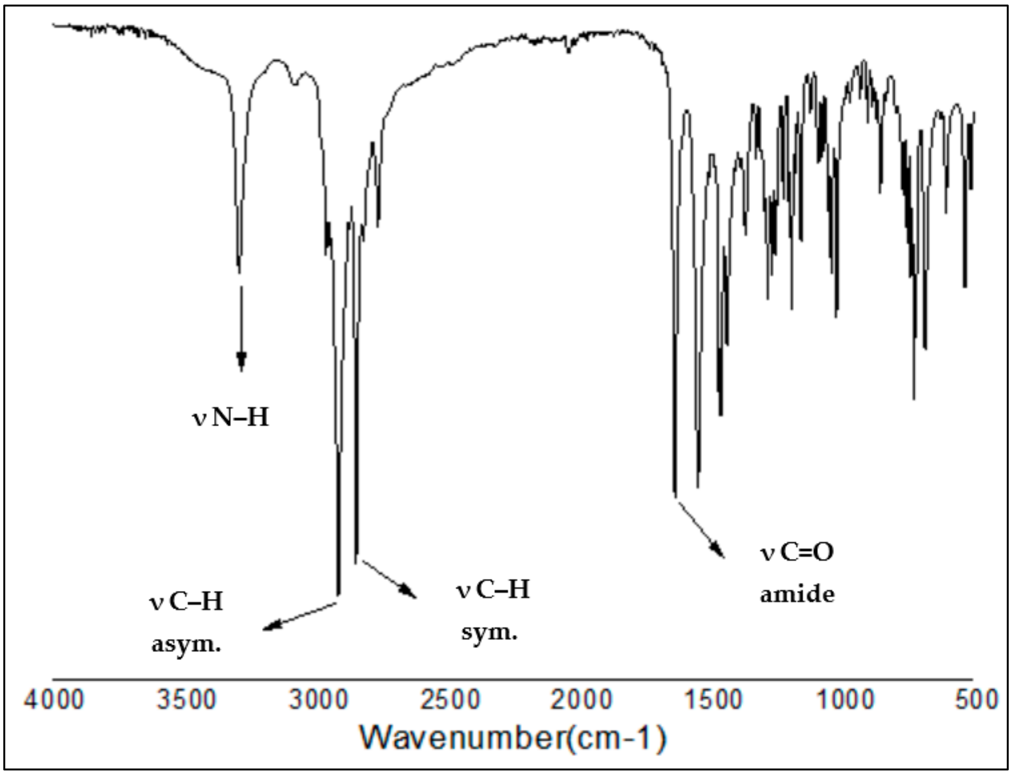

Figure S3 FT-IR Spectra for *DEAE16*.

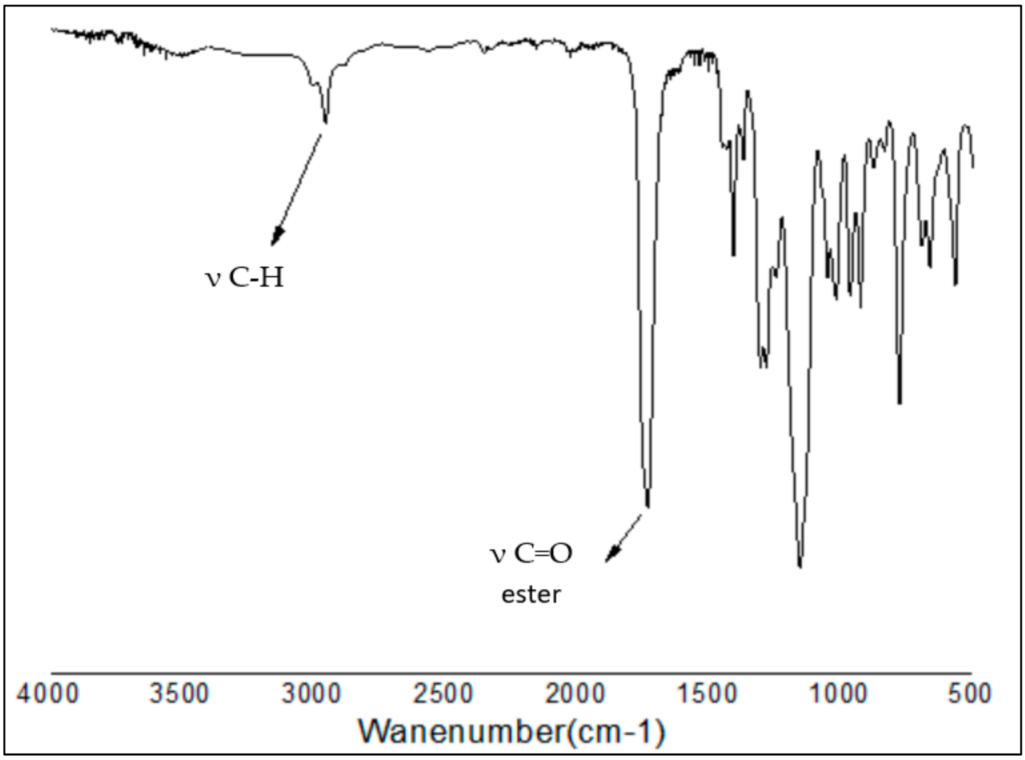

Figure S4 FT-IR Spectra for Ethane-1,2-diyl bis(2-chloroethanoat)

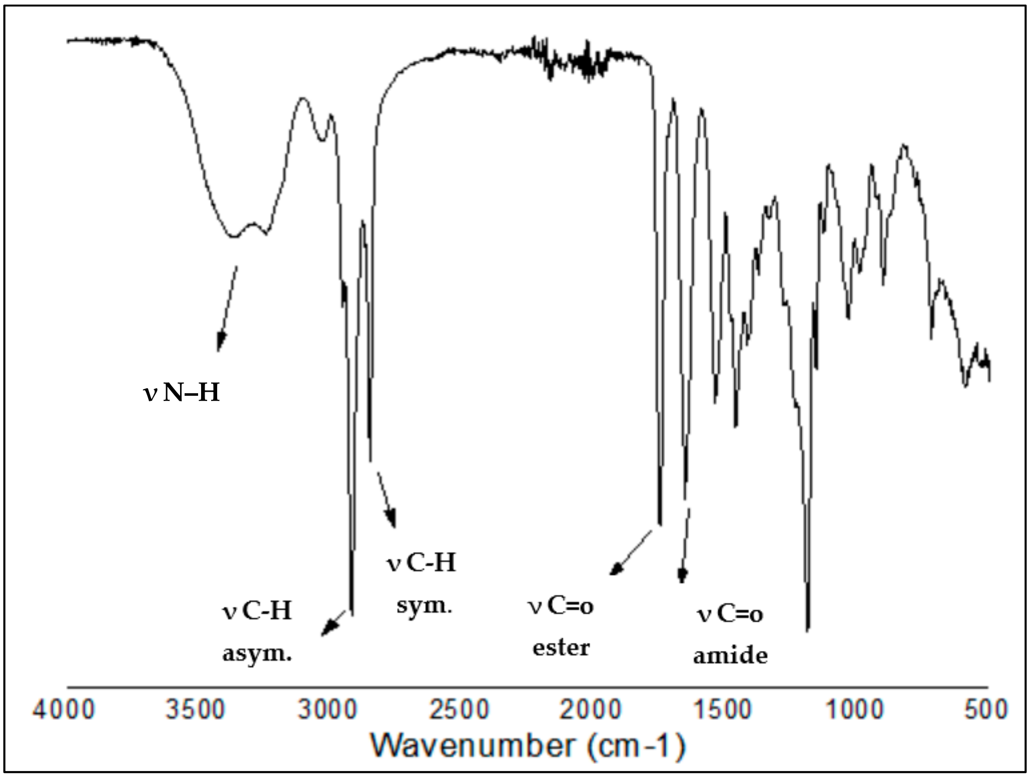

Figure S5 FT-IR Spectra for CGSES12.

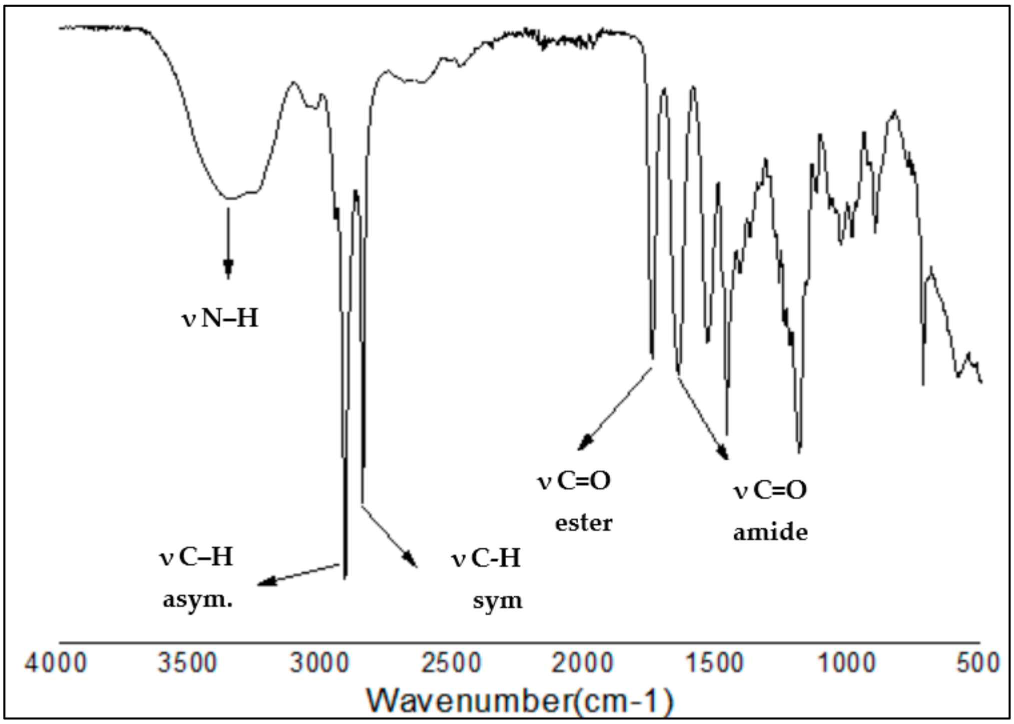

Figure S6 FT-IR Spectra for CGSES16.

1.1 Proton Nuclear Magnetic Resonance (^1H NMR)

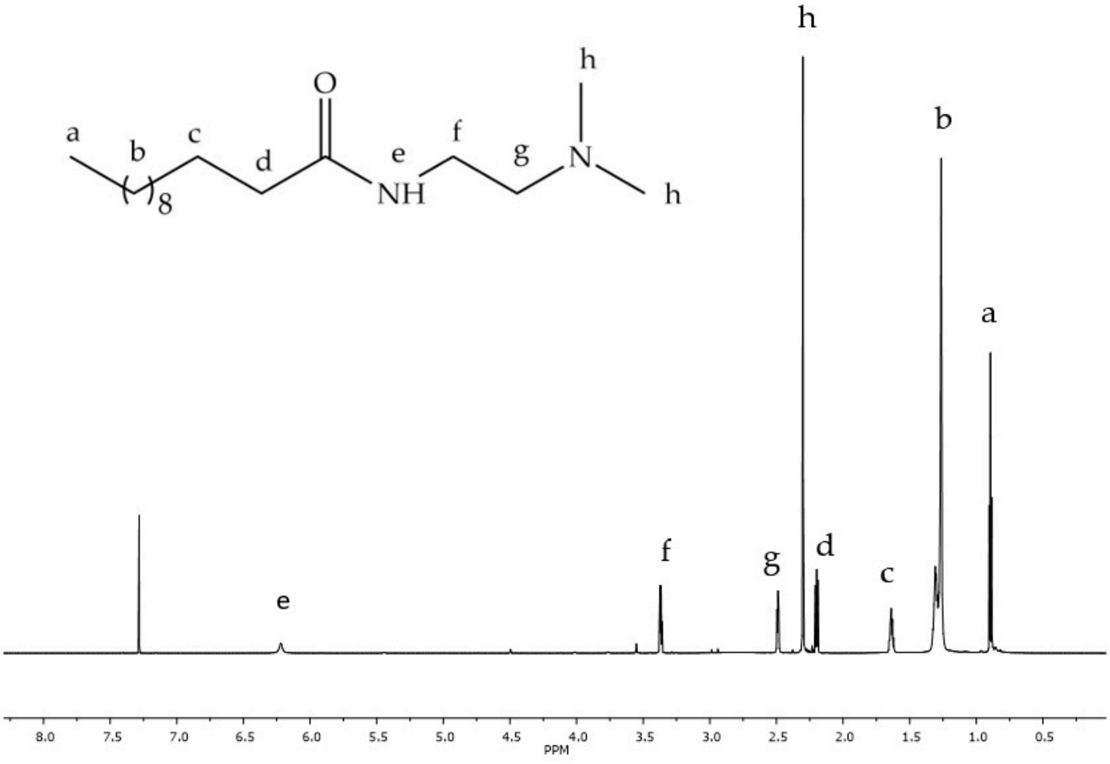

Figure S7 ^{1}H-NMR Spectra for DAEA12.

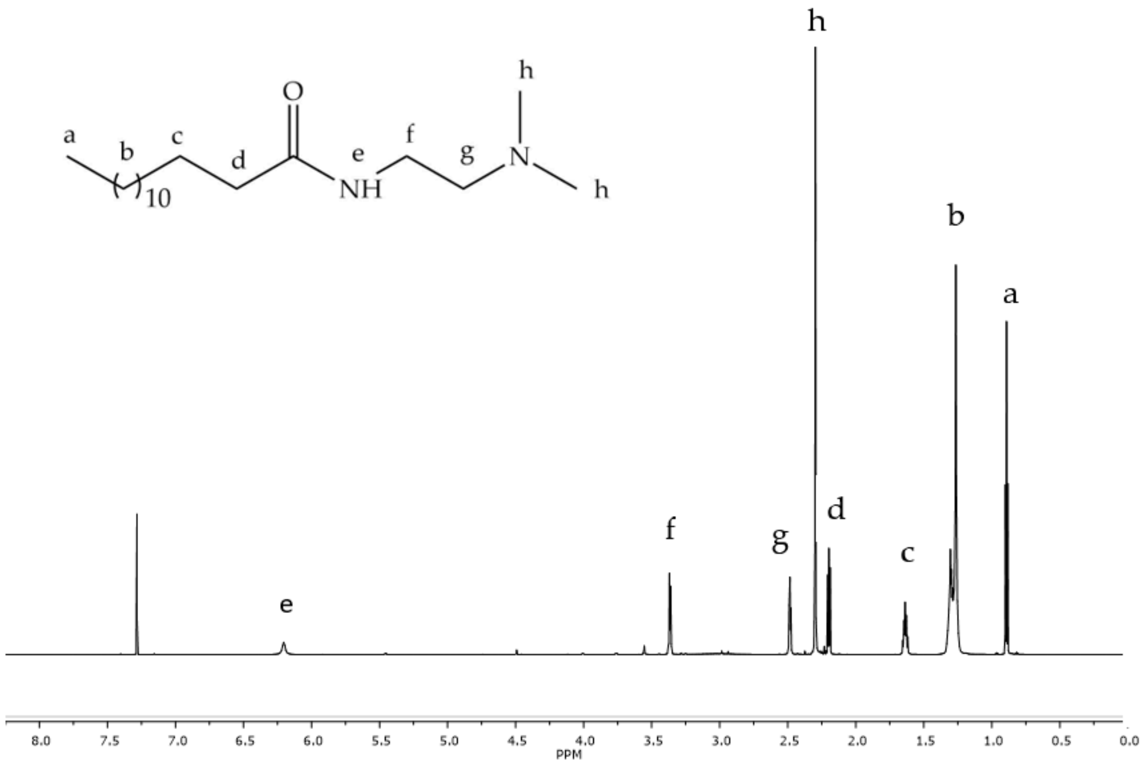

Figure S8 ^{1}H-NMR Spectra for DAEA14.

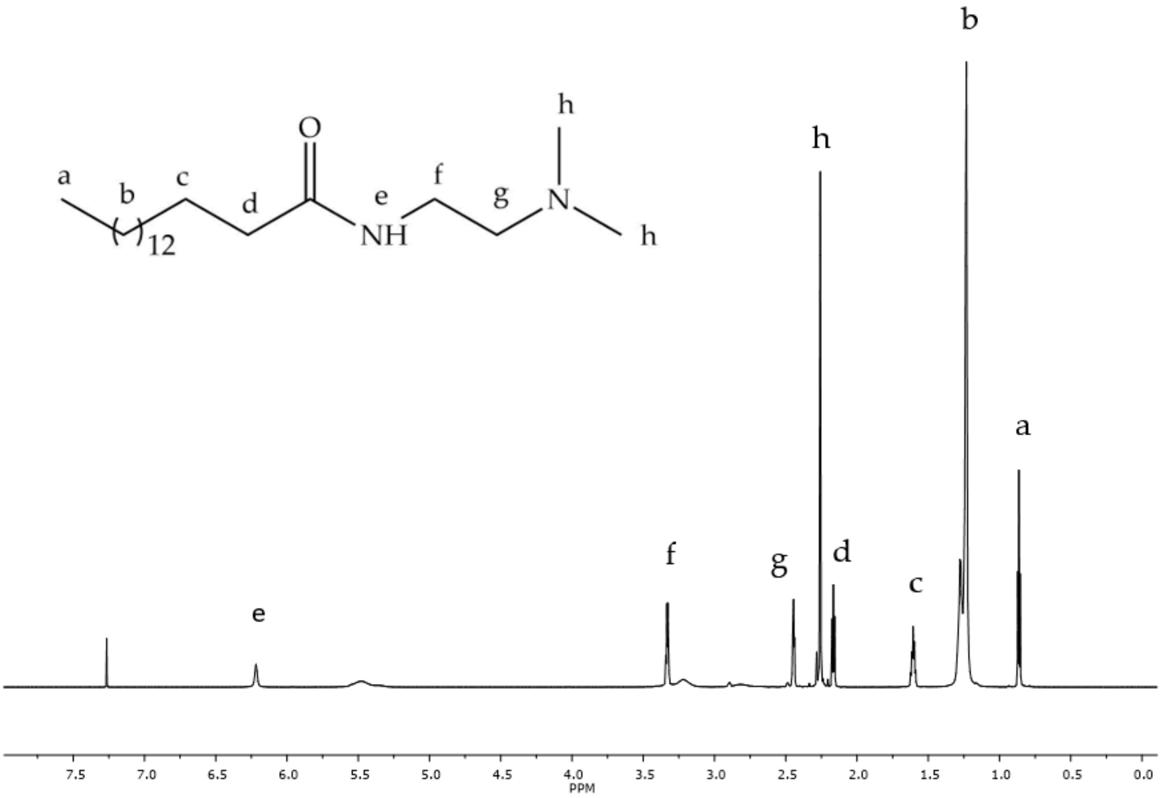

Figure S9 ^{1}H-NMR Spectra for DAEA16.

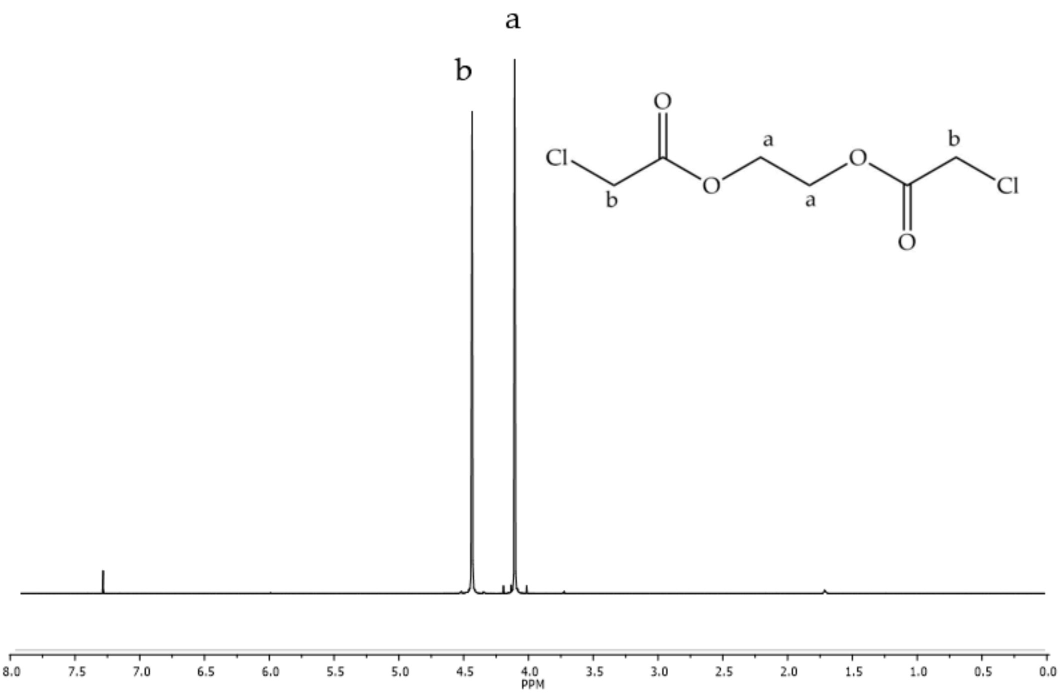

Figure S10 ^{1}H-NMR Spectra for Ethane-1,2-diyl bis(2-chloroethanoat).

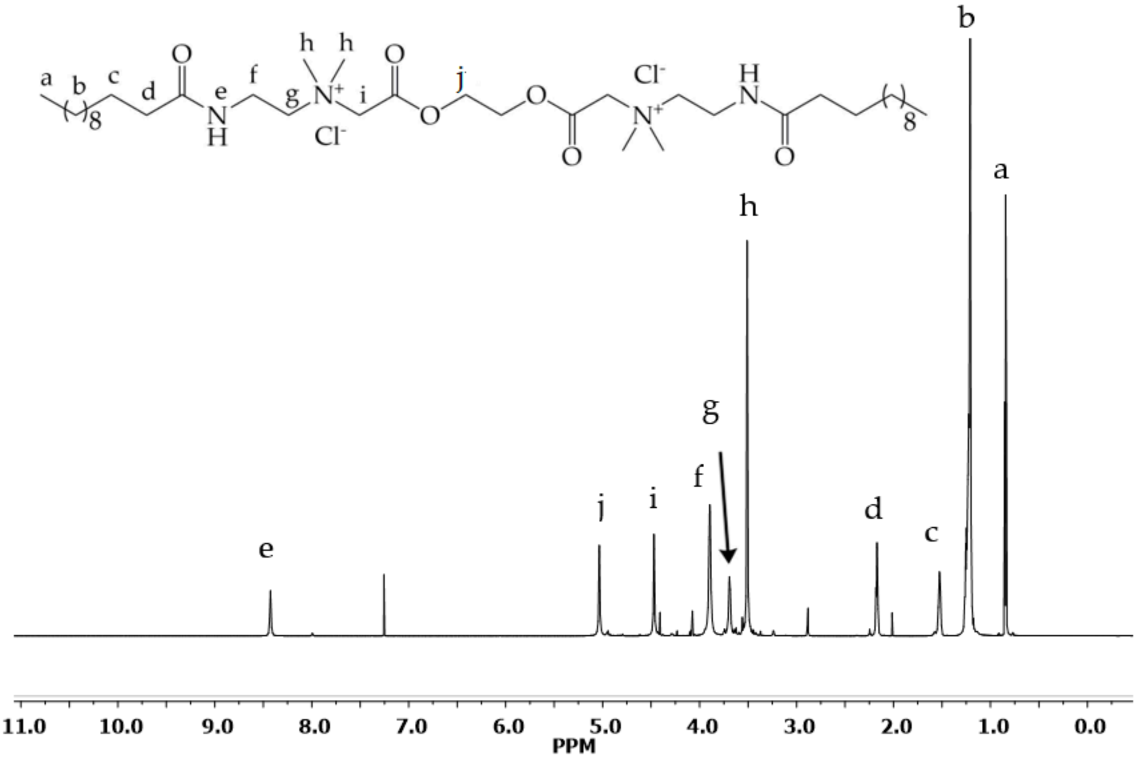

Figure S11 ^{1}H-NMR Spectra for CGSES12.

1.2 Nuclear magnetic resonance (^{13}C NMR)

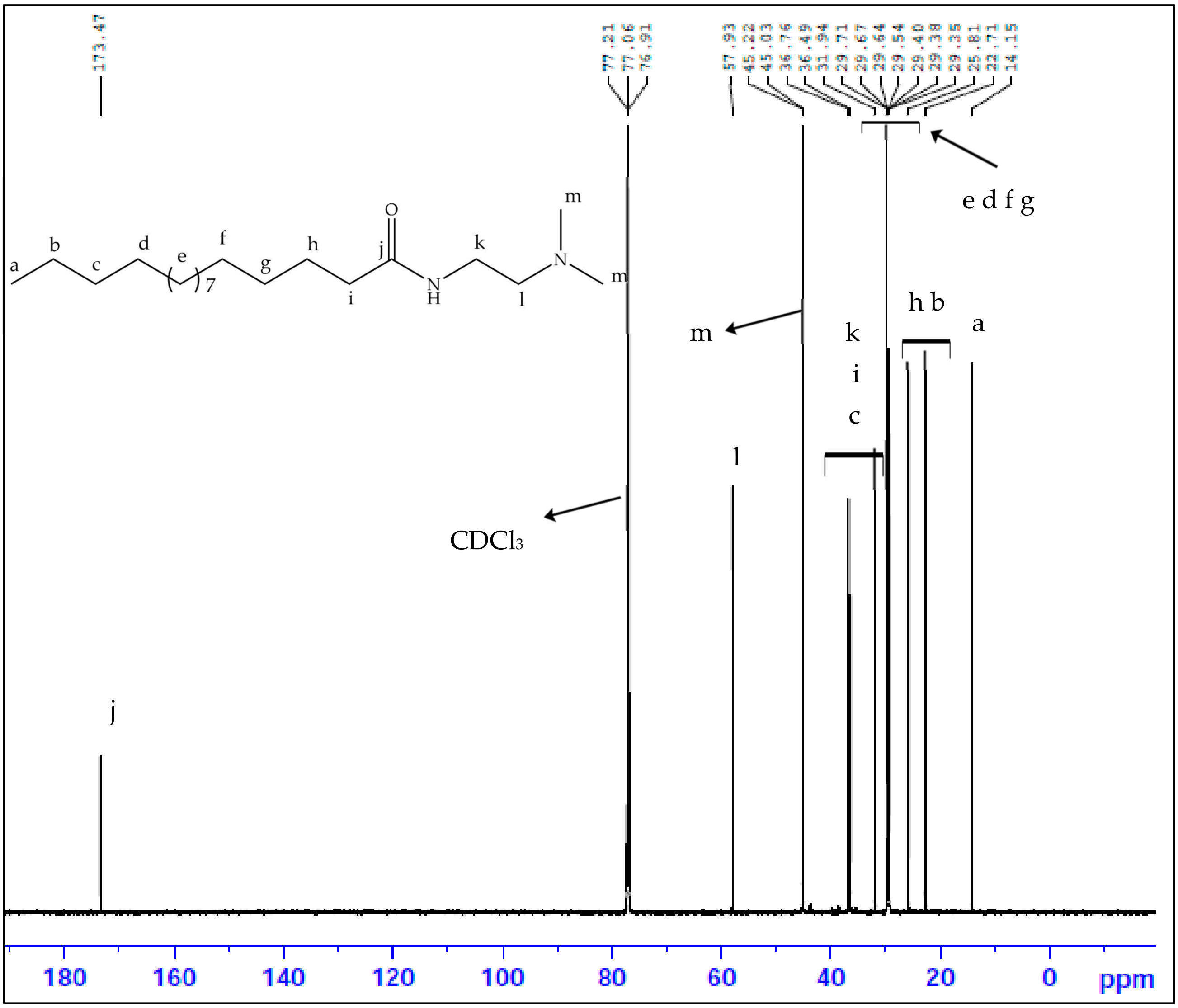

Figure S12 ^{13}C-NMR Spectra for DAEA16.

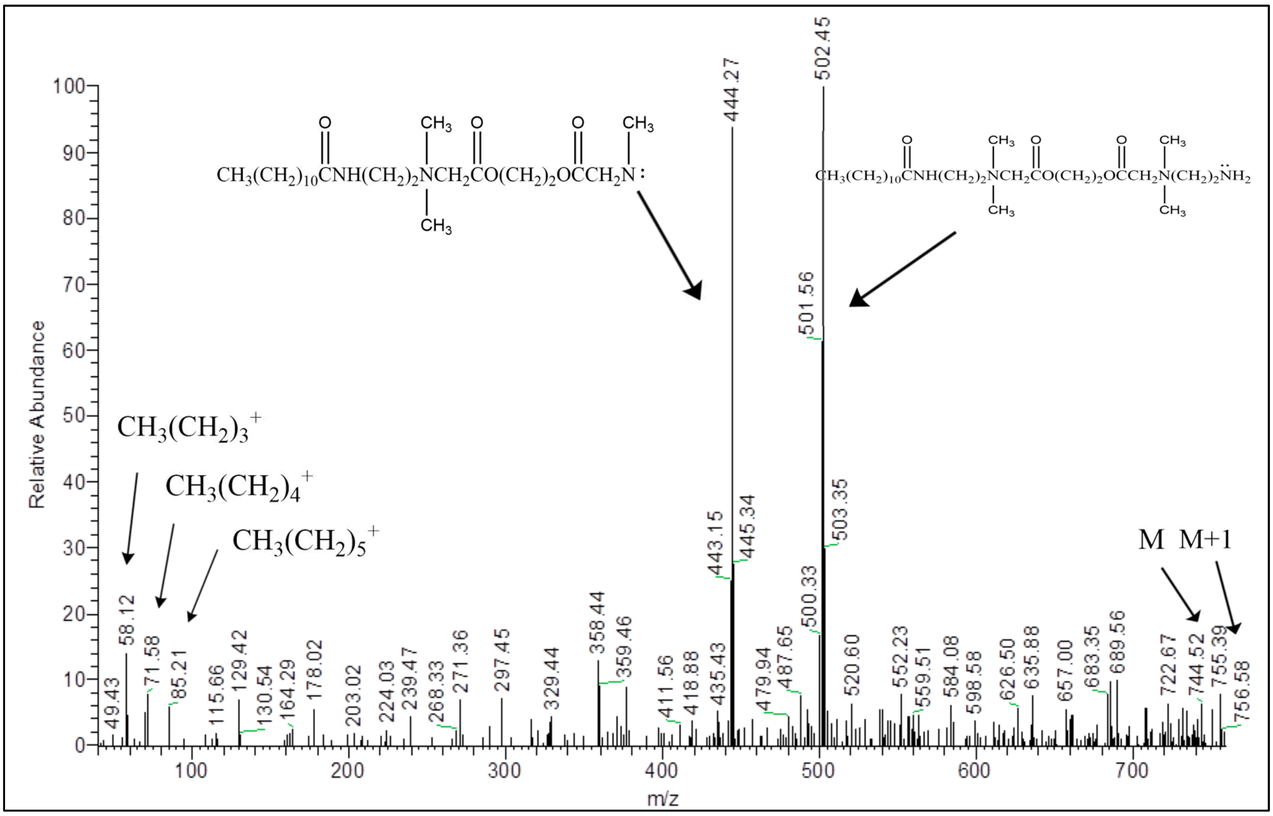

Figure S13 MassSpectra for CGSES12

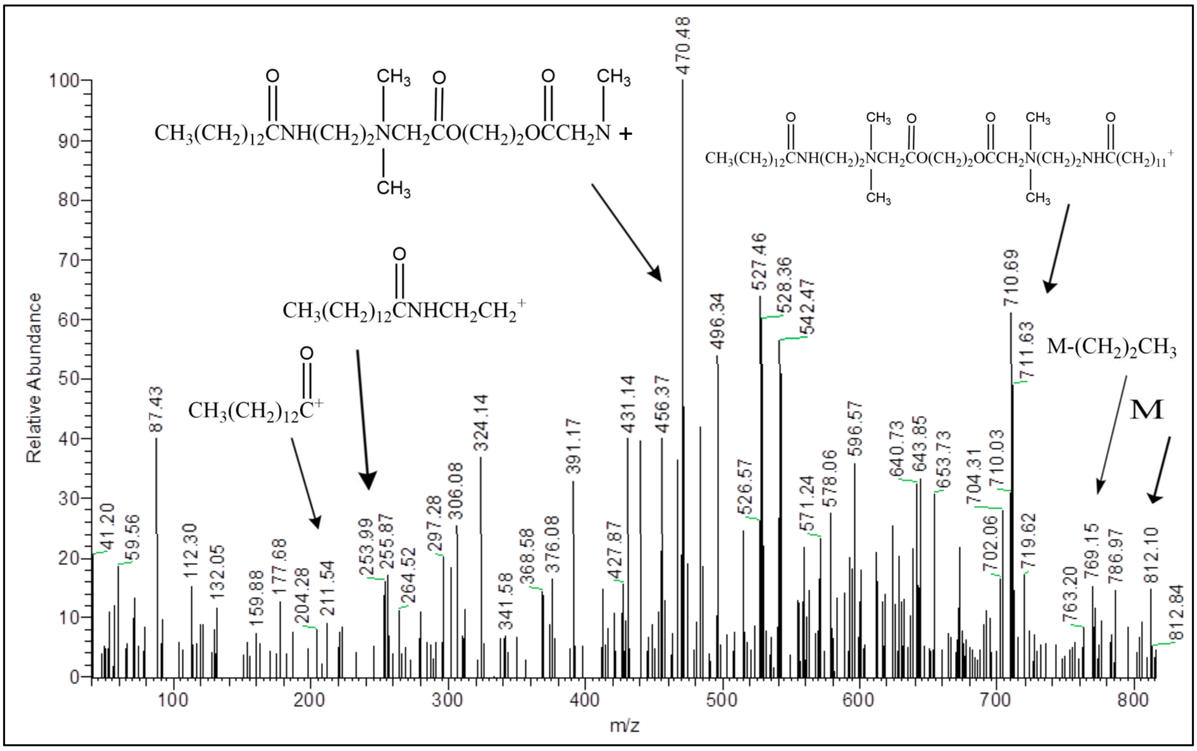

Figure S14 Mass Spectra for CGSES14.
